# Prevalence and genotypes’ distribution of human papillomavirus among women in Saudi Arabia: a systematic review and meta-analysis

**DOI:** 10.3389/fpubh.2025.1580699

**Published:** 2025-05-15

**Authors:** Mohammed S. Aldossary, Mohammed Mufrrih, Mervat M. El Dalatony, Hatoon M. Alamri

**Affiliations:** ^1^General Directorate of Research and Studies, Ministry of Health, Riyadh, Saudi Arabia; ^2^Department of Medical Laboratory Sciences, Faculty of Applied Medical Sciences, King Abdulaziz University, Jeddah, Saudi Arabia; ^3^Special Infectious Agents Unit BSL-3, King Fahd Medical Research Center, King Abdulaziz University, Jeddah, Saudi Arabia; ^4^Public Health and Community Medicine Department, Faculty of Medicine, Menofia University, Shebin Elkom, Egypt; ^5^Laboratory and Blood Bank Department, King Abdullah Medical City, Makkah, Saudi Arabia

**Keywords:** HPV infection, cervical cancer, genotype, Saudi Arabia, meta-analysis

## Abstract

**Introduction:**

Human Papillomavirus (HPV) is a prevalent sexually transmitted infection that can lead to benign lesions, premalignant changes, and cancer. Despite its significance, studies in Saudi Arabia report inconsistent findings regarding HPV prevalence and risk factors. This systematic review and meta-analysis aimed to assess the prevalence and genotype distribution of HPV among women in Saudi Arabia.

**Methods:**

A systematic literature search was conducted across multiple electronic databases (January 1990–August 2024). Studies reporting HPV prevalence among women in Saudi Arabia, regardless of nationality or health status, were included. The pooled prevalence was calculated using a random-effects model, with log-transformed proportions and 95% confidence intervals (CI).

**Results:**

Twenty-two studies (*n* = 15,224 women) met the inclusion criteria. The pooled prevalence of HPV among women attending cervical screening was 14.9% (95% CI: 10.9–18.9%), with substantial heterogeneity (I^2^ = 97.4%, *p* < 0.001). Subgroup analysis by region showed a higher prevalence in Riyadh (19.1, 95% CI: 13.1–25%) compared to the Western region (6.1, 95% CI: 3.7–8.4%). Among women with gynecological malignancies, the pooled prevalence was 68.1% (95% CI: 49–87.1%). HPV-16 was the most common genotype (35.4%), followed by HPV-18 (10.9%). Other high-risk types (HPV-45, 31, 33, 35, 52, and 58) accounted for 2.2–13.7% of infections.

**Conclusion:**

HPV prevalence in Saudi Arabia is comparable to global figures, though significant geographic variability exists. A national screening survey is necessary to establish the true prevalence and inform preventive strategies.

**Systematic Review Registration:**

PROSPERO No. CRD42024583260.

## 1 Introduction

Human papillomavirus (HPV) is one of the most common sexually transmitted infections globally, with over 85% of sexually active individuals estimated to contract HPV at some point in their lives (1). Currently, approximately 300 million women worldwide have an active HPV infection. The global prevalence of HPV infection among women with normal cervical cytology is approximately 9.9%, while HPV carrier prevalence in this group is around 32.1% (2, 3). However, the epidemiology of HPV varies significantly between regions, influenced by factors such as sexual behavior, healthcare access, vaccination programs, and cultural practices. Epidemiological data show that HPV prevalence among women with normal cytology is highest in United States (38.4%), Sub-Saharan Africa (24%), and Europe (11–12%) (3–5). HPV is primarily transmitted through direct sexual skin-to-skin contact or, less commonly, during non-sexual routes (6, 7). The incubation period for HPV varies, ranging from weeks to several months after exposure, with many infections remaining asymptomatic. Clinical manifestations depend on the HPV genotype, with low-risk types causing benign warts and high-risk types potentially leading to premalignant and malignant lesions (8, 9). Signs and symptoms of HPV-related diseases include genital warts, respiratory papillomatosis, and lesions that may progress to cervical, oropharyngeal, or other anogenital cancers in high-risk infections (8, 10).

Currently, there are more than 200 identified HPV genotypes, of which approximately 40 can infect the genital area (11). HPV is classified into low-risk and high-risk types based on their association with cervical cancer and precursor lesions. Among the high-risk genotypes, HPV-16 and HPV-18 are the most prevalent, accounting for approximately 70% of all cervical cancer cases globally (12). While most HPV infections are transient and resolve within 2 years, some infections persist and may progress to benign lesions, premalignant lesions, and cancer (13, 14). Low-risk HPV can lead to genital warts or papilloma; on the other hand, high-risk HPV infections are well-recognized causes of pre-cancerous lesions and, ultimately, invasive cervical carcinoma. HPV is also implicated in the anal, vulvar, vaginal, and penile cancers (13, 15). Several risk factors have been identified that increase the likelihood of persistent HPV infection, including multiple sexual partners, unprotected sex, high parity, immunosuppression, smoking, and long-term use of combined oral contraceptives (COCs) (16, 17). The prevalence of COCs use in Saudi Arabia was reported to be 24.4% (18), compared to a global figure of 8% (19).

Given the crucial role of HPV infection in cervical cancer, HPV screening and vaccination have become pivotal components of cervical cancer prevention strategies, reducing the incidence of cervical cancer and its associated morbidity and mortality (20). In Saudi Arabia, current data suggest an HPV prevalence ranging from 4.3 to 43%, with high-risk genotypes accounting for most cases (21, 22). These alarming figures are accompanied by significant increases in the incidence and mortality of HPV-related malignancies over the past few decades (23). Nearly 70% of women with cervical cancers in Saudi Arabia are diagnosed at later stages and have never undergone HPV screening (24, 25). Epidemiological studies from Saudi Arabia are also limited by the small sample sizes and the lack of multi-center evaluation, underestimating the actual burden of HPV infection in the Kingdom. Thus, it is crucial to understand the actual prevalence of HPV infection as a first step toward establishing a comprehensive national screening and vaccination program. The present systematic review and meta-analysis evaluated the prevalence and genotypes’ distribution of HPV infection among women in Saudi Arabia.

## 2 Methods

We prepared the present manuscript in concordance with the PRISMA (Preferred Reporting Items for Systematic Reviews and Meta-Analyses) 2020 checklist (26). The review protocol was registered on the PROSPERO register for systematic review protocols (CRD42024583260).

### 2.1 Eligibility criteria

We included published reports that fulfilled the following inclusion criteria: (1) studies that included adult women living in Saudi Arabia who were screened for HPV infection, regardless of their health status, nationality, ethnicity, or socio-economic status; (2) studies that assessed the presence of HPV infection using validated laboratory diagnostic methods, such as polymerase chain reaction (PCR) testing, DNA hybridization assays, HPV genotyping techniques, or other validated molecular methods; (3) studies that focused on detecting HPV DNA in cervical, vaginal, or other genital samples; (4) studies that reported at least the prevalence or genotype distribution of HPV infection among the study population; and (5) studies that were observational studies, including retrospective chart reviews, cohort studies, cross-sectional studies, and case–control studies. We excluded unpublished studies -including conference abstracts, preprints, and theses-, review articles, case reports, and in-vitro studies. Studies that were published in languages other than English were excluded as well.

### 2.2 Information source, search strategy, and selection process

A systematic literature search was conducted across multiple electronic databases from January 1990 to August 2024, including Medline via PubMed, EMBASE via Ovid, Web of Science, Scopus, and CINAHL (Cumulative Index to Nursing and Allied Health Literature). A combination of Medical Subject Headings (MeSH) terms, free-text keywords, and Boolean operators were employed. The primary search terms included “Human Papillomavirus,” “HPV,” “prevalence,” “genotype,” and “Saudi Arabia.” The search was not restricted by publication year to include all relevant literature. Additionally, reference lists of included studies were screened to identify any additional articles. The detailed search strategy, including the list of search terms and the number of articles retrieved from each database, is provided in Supplementary Table 1.

Two reviewers performed the screening process independently, and discrepancies were resolved through discussion and consensus. All retrieved records were initially imported to Rayyan software,^1^ and duplicates were removed. Unique records were screened in two stages: title and abstract screening, followed by full-text review.

### 2.3 Data collection process and risk of bias assessment

A standardized data extraction form was developed to collect relevant data from the included studies. Two independent reviewers extracted the data, and any disagreement was resolved by consensus. We extracted the study design, data collection window, studied population, sample size, diagnostic methods, specimens, baseline characteristics of the participants, the prevalence of HPV infection, the distribution of HPV genotypes, and the histopathological distribution of samples according to the HPV genotyping. The primary outcome of the present study was the pooled prevalence of HPV infection, defined as the proportion of women in the study population who test positive for any type of HPV infection, as determined by molecular diagnostic methods. When multiple diagnostic tests were used, we considered the PCR-confirmed prevalence. The secondary outcomes included the incidence of HPV infection, genotypes’ distribution, clinical characteristics of women with HPV infections, risk factors for HPV positivity, and the association between HPV infection and cancer incidence.

The risk of bias in the included studies was assessed independently by two reviewers. For cohort and retrospective studies, the Newcastle-Ottawa Scale (NOS) was used (27). This scale evaluates studies based on three key domains: selection of participants, comparability of study groups, and outcome assessment. For the cross-sectional studies, the NOS by Herzog et al. was adopted (28). The overall risk of bias was categorized into low (score of 7–9), moderate (score of 4–6), or high (score of 0–3) risk of bias (27).

### 2.4 Statistical analysis

All statistical analyses were performed using OpenMeta [Analyst] (29). The pooled prevalence of HPV and the distribution of genotypes were calculated using a random-effects model to account for heterogeneity among the included studies. Proportions were log-transformed, and pooled estimates were presented along with 95% confidence intervals (CI). The statistical heterogeneity was evaluated using the visual inspection of the forest plot, the Chi^2^-test (significant if *p* < 0.10), and the I^2^ statistic; the I^2^ statistic quantifies the proportion of variability in effect estimates due to heterogeneity, with an I^2^ value ≥50% was considered indicative of substantial heterogeneity. If high heterogeneity was detected, a leave-one-out sensitivity and subgroup analyses were performed based on the studied population, the geographical distribution, or diagnostic methods. To further explore sources of heterogeneity, a meta-regression analysis was conducted using study-level covariates, including mean/median age. These variables were selected based on availability and consistency across the included studies.

## 3 Results

**Figure 1:**
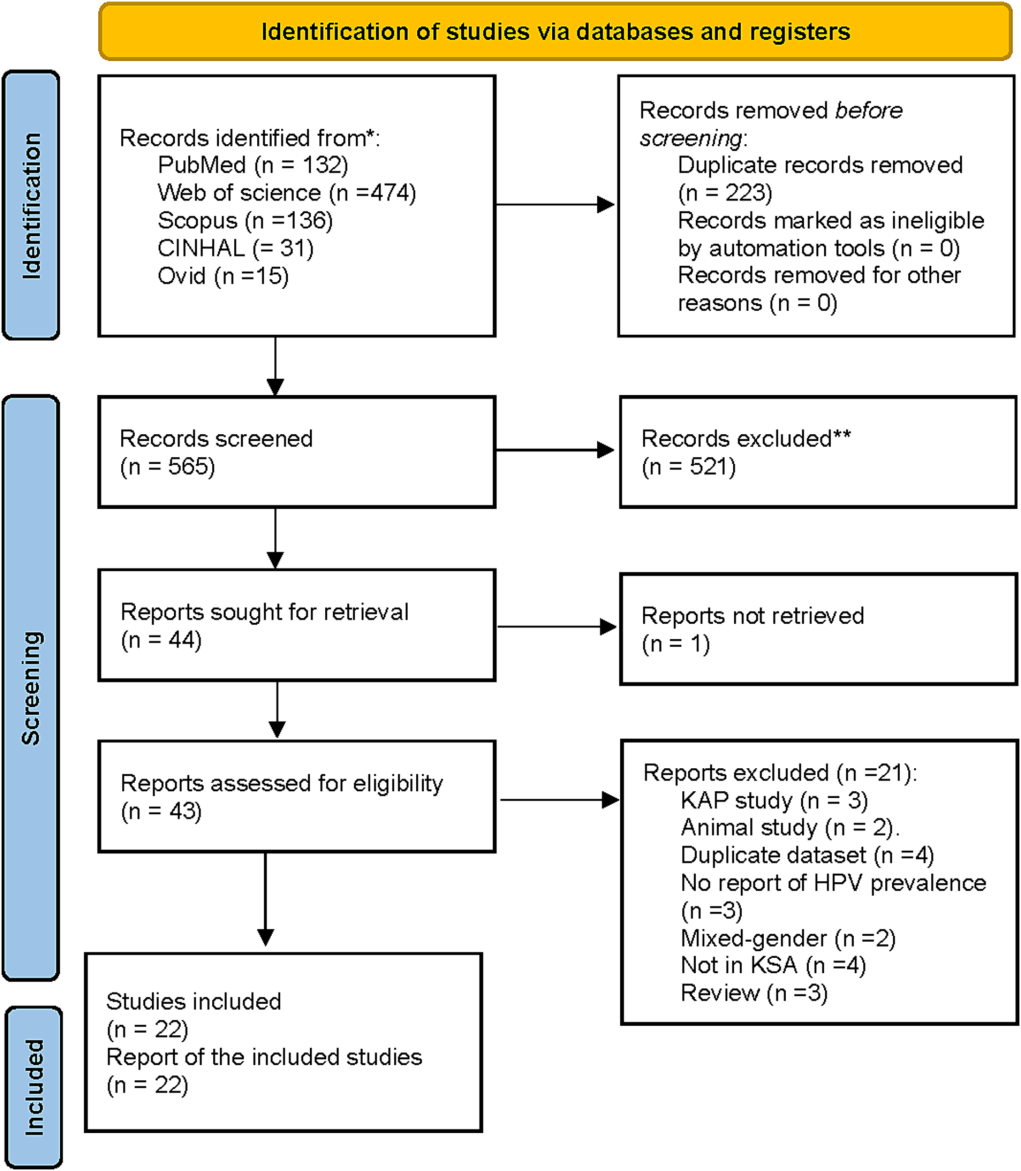
PRISMA flowchart.

A total of 788 records were identified. After duplicate removals, 565 unique records were screened based on titles and abstracts, and 43 full texts were retrieved to assess eligibility. Of them, 21 reports were excluded due to being knowledge, attitude, and practice (KAP; *n* = 3), animal studies (*n* = 2), duplicate datasets (*n* = 4), studies with no report on HPV prevalence (*n* = 3), mixed-gender studies (*n* = 2), studies not conducted in Saudi Arabia (*n* = 4), and review articles (*n* = 3). A total of 22 studies (*n* = 15,224 women) met the eligibility criteria and were included in this systematic review and meta-analysis (21, 22, 30–49) (Figure 1).

### 3.1 Characteristics of the included studies

The summary characteristics of the included studies are summarized in Table 1. The majority of the included studies were retrospective analyses (*n* = 10) or cross-sectional studies (*n* = 9). Most studies were single-center experiences conducted in tertiary care centers, and the data collection window spanned over two decades (1990–2019). In terms of the studied population, the majority of the included studies (*n* = 15) included adult women who underwent routine cervical cancer screening (21, 22, 31, 32, 34–38, 40–42, 44, 48, 49), while five studies included women with cervical or ovarian cancers (39, 43, 45–47). The remaining two studies focused on women with abnormal cytology (30, 33). The sample sizes varied considerably, from 40 to 5,360 participants. The diagnostic methods used to detect HPV varied among the included studies. The most frequently used methods were real-time PCR (*n* = 10), followed by nested PCR (*n* = 6), Hybrid Capture 2 (HC2; *n* = 3), and Hybrid PCR (*n* = 3). The analyzed specimen included predominantly cervical swabs or cytology (21, 22, 31, 32, 34–38, 40–42, 44, 48, 49).

**Table 1:** Summary characteristics of the included studies (*n* = 22 studies).

Study ID	Study design	Study duration	Population	Setting	Region	Purpose of sample collection	Sample size	Type of specimen	Method of diagnosis	Main findings	Overall RoB
Sait et al. 2024 (21)	A multi-center combined cross-sectional and prospective study	From 2013 to 2018	Ever-married women aged 30–65	Tertiary and primary care centers	Jeddah	Cervical cancer screening	5,360	Cervical swabs	HC2 HPV test	HPV prevalence is low but requires continuous monitoring	Low
Faqih et al. 2023 (30)	A single-center retrospective analysis	From 2021 to 2022	Women aged 23 to 82 with abnormal cytology	Tertiary care center	Riyadh	Cervical cancer screening	155	Cytology brushes	Real-time PCR	The association between specific HPV genotypes and cervical abnormalities is controversial and requires further evidence.	Moderate
Alshammari et al. 2022 (31)	A single-center cross-sectional study	From 2020 to 2021	Saudi women aged 20 to 70 years with gynecologic complaints	Tertiary care center	Al-Madinah	Cervical cancer	300	Exfoliative cytology	PCR	High-risk HPV infection is low. Cervical abnormalities are associated with HPV infection.	Low
Alhamlan et al. 2021 (32)	A single-center retrospective analysis	From 2006 to 2016	Women aged 23–95 years old	Tertiary care center	Riyadh	Cervical cancer	315	FFPE cervical biopsy	Real-time PCR	HPV screening is important to reduce the risk of cervical cancer	Moderate
Kussaibi et al. 2021 (33)	A single-center retrospective analysis	From 2013 to 2019	Saudi women with ASCUS coinvestigated for HR HPV along with Pap tests	Tertiary care center	Eastern Province	Cervical cancer	164	Cytology brushes	Real-time PCR	There is a geographical difference in the HR HPV frequency and genotype distribution.	Moderate
AlBabtain et al. 2020 (34)	A single-center retrospective analysis	From 2002 to 2017	Women aged 21 and 65	Tertiary care center	Riyadh	Cervical Cancer	3,346	Pap smears	PCR	HPV prevalence is low but requires continuous monitoring	High
Alhamlan et al. 2020 (35)	A single-center retrospective analysis	NA	Ever married women	Tertiary care center	Riyadh	Cervical cancer	608	Cytology brushes	PCR	HPV prevalence is comparable to other reports from Riyadh	Moderate
Obeid 2020 et al. (36)	A single-center retrospective analysis	NA	Women who underwent cervical screening	Tertiary care center	Riyadh	Cervical cancer	933	FFPE cervical biopsy and Pap smears	Nested-PCR	HPV load is a predictor of cervical cancer	Moderate
Ali et al. 2019 (37)	A multi-national cross-sectional study	NA	Women residing in Saudi Arabia	Multiple centers	Across Saudi Arabia	Cervical cancer	1,276	Cytology brushes	Real-time PCR	The study supports national screening and vaccination programs	Low
Mousa et al. 2019 (38)	A single-center cross-sectional study	From 2017 to 2018	Married women > 18 years	Tertiary care center	Jeddah	Cervical cancer	119	Vaginal swabs	Nested-PCR	HPV prevalence is low, with higher prevalence among high-risk groups	Low
Alsbeih et al. 2017 (39)	A single-center retrospective analysis	From 1990 to 2012	Women with invasive cervical cancer	Tertiary care center	Riyadh	Cervical cancer	232	Histopathological slides or blood samples	Nested-PCR	HPV screening may be useful in cervical cancer	Moderate
Alhamlan et al. 2016 (40)	A multi-center cross-sectional study	From 2013 to 2015	Women undergoing routine cervical examinations	Tertiary and primary care centers	Riyadh	Cervical cancer	400	Cytology brushes	Hybrid PCR	There is a high prevalence of HPV infection	Low
Al-Ahdal et al. 2014 (41)	A single-center cross-sectional study	NA	Women residing in Saudi Arabia	Tertiary care center	Riyadh	Cervical cancer	519	Cytology brushes	Nested-PCR	HPV is common among women in Riyadh	Moderate
AlObaid et al. 2014 (42)	A multi-center cross-sectional study	From 2010 to 2011	Women undergoing routine cervical examinations	Multiple centers	Riyadh	Cervical cancer	417	Cytology brushes	Nested-PCR	HPV prevalence was relatively low	Low
Al-Shabanah et al. 2013 (43)	A single-center retrospective analysis	NA	Women with ovarian cancer	Tertiary care center	Riyadh	Ovarian carcinoma	100	FFPE biopsy	Nested-PCR	HPV may have a role in ovarian carcinogenesis	High
Bondagji et al. 2013 (44)	A single-center cross-sectional study	From 2010 to 2011	Saudi women of different age groups attending gynecology clinic	Tertiary care center	Jeddah	Cervical cancer	485	Cervical scrapes	Hybrid PCR	HPV prevalence was relatively low	Moderate
Turki 2013 et al. (22)	A single-center cross-sectional study	From 2011 to 2012	Women with gynecological complaints	Tertiary care center	Jeddah	Cervical cancer	40	Tissue biopsies	Hybrid PCR	HPV prevalence is alarmingly increasing.	Moderate
Al-Badawi et al. 2011 (45)	A single-center retrospective analysis	From 1997 to 2007	Women with cervical cancer and carcinoma *in situ*	Tertiary care center	Riyadh	Cervical Cancer	90	FFPE biopsy	PCR	HPV prevalence in cervical cancer patients is comparable to other international report	Moderate
Alsbeih et al. 2011 (46)	A single-center retrospective analysis	NA	Women with cervical tumors	Tertiary care center	Riyadh	Cervical cancer	100	FFPE biopsy	PCR	HPV16-18 was associated with earlier onset of cervical cancer	High
Sait 2011 et al. (47)	A single-center cross-sectional study	From 2007 to 2008	Women with cervical cancer and carcinoma in situ	Tertiary care center	Jeddah	Cervical dysplasia and invasive disease	45	Cervical biopsy	HC2 HPV test	HPV infection predisposes to cervical cancer	Moderate
Al-Muammar et al. 2007 (48)	A single-center prospective study	NA	Women attending a family medical clinic	Primary care center	Riyadh	Cervical Cancer	120	Cervical scrapes (both ectocervical and endocervical)	PCR	Despite the high prevalence, HPV 16/18 does not contribute to the progression of CIN	Moderate
Gazzaz et al. 2007 (49)	A multi-center prospective study	2006	Women who underwent cervical screening	Tertiary care centers	Jeddah	Cervical cancer	100	Cytology brushes	HC2 HPV test	Combined screening by cytology and HPV testing detects women with existing disease	High

ASCUS, Atypical Squamous Cells of Undetermined Significance; CIN, Cervical Intraepithelial Neoplasia; DNA, Deoxyribonucleic Acid; FFPE, Formalin-Fixed, Paraffin-Embedded; HC2, Hybrid Capture 2; HPV, Human Papillomavirus; HR HPV, High-Risk Human Papillomavirus; NA, Not Available; PCR, Polymerase Chain Reaction; RoB, Risk of Bias.

Concerning the baseline characteristics (Supplementary Table 2), the majority of participants were between 31 and 50 years. Among studies that recruited diverse nationalities, 55–86% of the women were Saudi nationals, while few participants had no formal education (range 5.3–6.5%). On the other hand, 63% had studied up to post-secondary/university level (42). Most women (74.6–100%) were married at the time of data collection. Few studies reported the smoking status and the use of COCs; the percentages of current smokers and COCs use were relatively low. The histopathological findings of participants were variable across studies. Among studies that included women undergoing routine cervical screening, the rate of atypical squamous cells, cannot exclude high-grade lesions (ASC-H) or higher grades ranged from 1 to 27.5%.

### 3.2 Risk of bias

Most cross-sectional studies were rated as having a low or moderate risk of bias (see Table 1). All cross-sectional studies had well-defined selection criteria and participant recruitment methods. However, only a few studies, like Sait et al. (21), Ali et al. (37), and Mousa et al. (38), controlled for key factors, such as age and cytological status. Most included cross-sectional studies also provided detailed descriptions of statistical methods for outcome assessment (Supplementary Table 3). On the other hand, one prospective study (49) and three retrospective studies (34, 43, 46) were rated as high risk of bias, primarily due to a lack of control for confounding factors, inadequate ascertainment of exposure, or insufficient justification for participant selection. The remaining prospective and retrospective studies had a moderate risk of bias (Supplementary Tables 3, 4).

### 3.3 Prevalence and incidence of HPV infection

The prevalence of HPV infection among women in Saudi Arabia is presented in Table 2.

**Table 2:** Prevalence and genotype distribution of HPV (*n* = 22 studies).

Study ID	No	Prevalence of HPV	HPV genotype				
			HPV-16	HPV-18	HPV-45	HPV types 31, 33, 35, 52, and 58	Combined or Others
General population							
Sait et al. 2024 (21)	5,360	254 (4.3%)	NA	NA	N/A	N/A	N/A
Alshammari et al. 2022 (31)	300	14 (4.6%)	6 (42.8%)	1 (7%)	0	7 (50%)	0
Alhamlan et al. 2021 (32)	315	96 (30.47%)	54 (56.3%)	7 (7.3%)	1 (1.0%)	11 (11.5%)	23 (23.9%)
AlBabtain et al. 2020 (34)	274	18 (6.6%)	3 (18.8%)	1 (5.5%)	N/A	2 (11.1%)	12 (66.7%)
Alhamlan et al. 2020 (35)	608	69 (11.4%)	9 (18%)	27 (54%)	0	2 (4%)	31 (20%)
Obeid et al. 2020 (36)	933	165 (17.7%)	62 (51.2%)	34 (28.1%)	0	7 (5.8%)	18 (14.8%)^*^
Ali et al. 2019 (37)	1,276	219 (17.2%)	47 (21.5%)	5 (2.3%)	N/A	124 (56.6%)	43 (19.6%)
Mousa et al. 2019 (38)	119	7 (5.9%)	NA				
Alhamlan et al. 2016 (40)	400	67 (17%)	13 (19.4%)	23 (34%)	0	7 (10.4%)	24 (35.8%)
Al-Ahdal et al. 2014 (41)	519	164 (31.6%)	N/A				
AlObaid et al. 2014 (42)	417	41 (9.8%)	3 (7.3%)	4 (9.8%)	0	6 (14.6%)	28 (68.3%)
Bondagji et al. 2013 (44)	485	27 (5.6%)	NA				
Turki et al. 2013 (22)	40	17 (43%)	12 (30%)	3 (7.5%)	2 (5%)	0	0
Al-Muammar et al. 2007 (48)	120	38 (31.6%)	16 (42%)	4 (11.1%)	0	0	18 (47.4%)
Gazzaz et al. 2007 (49)	100	6 (6%)	NA				
Women with cytological abnormalities							
Faqih et al. 2023 (30)	155	82 (52.9%)	18 (11.6%)	6 (3.9%)	6 (3.9%)	N/A	52 (31%)
Kussaibi et al. 2021 (33)	164	24 (16.4%)	8 (33.3%)	2 (8.3%)	N/A	N/A	14 (58.3%)
Gynecological malignancies							
Alsbeih et al. 2017 (39)	232	163 (77%)	110 (67.5%)	11 (6.8%)	9 (5.5%)	9 (5.5%)	N/A
Al-Shabanah et al. 2013 (43)	100	42 (42%)	18 (42.9%)	11 (26.2%)	3 (7.1%)	0	10 (23.8%)
Al-Badawi et al. 2011 (45)	90	86 (95.5%)	57 (63.4%)	10 (11.1%)	4 (4.5%)	9 (10.5%)	6 (6.9%)
Alsbeih et al. 2011 (46)	100	89 (89%)	58 (65.2%)	3 (3.4%)	6 (6.7%)	7 (7.9%)	15 (16.9%)
Sait et al. 2011 (47)	45	18 (47.4%)	NA				

HPV, Human Papillomavirus; NA, Not Available. *Data regarding the genotypes were not available for 44 patient.

#### 3.3.1 HPV prevalence among women attending routine cervical screening

**Figure 2:**
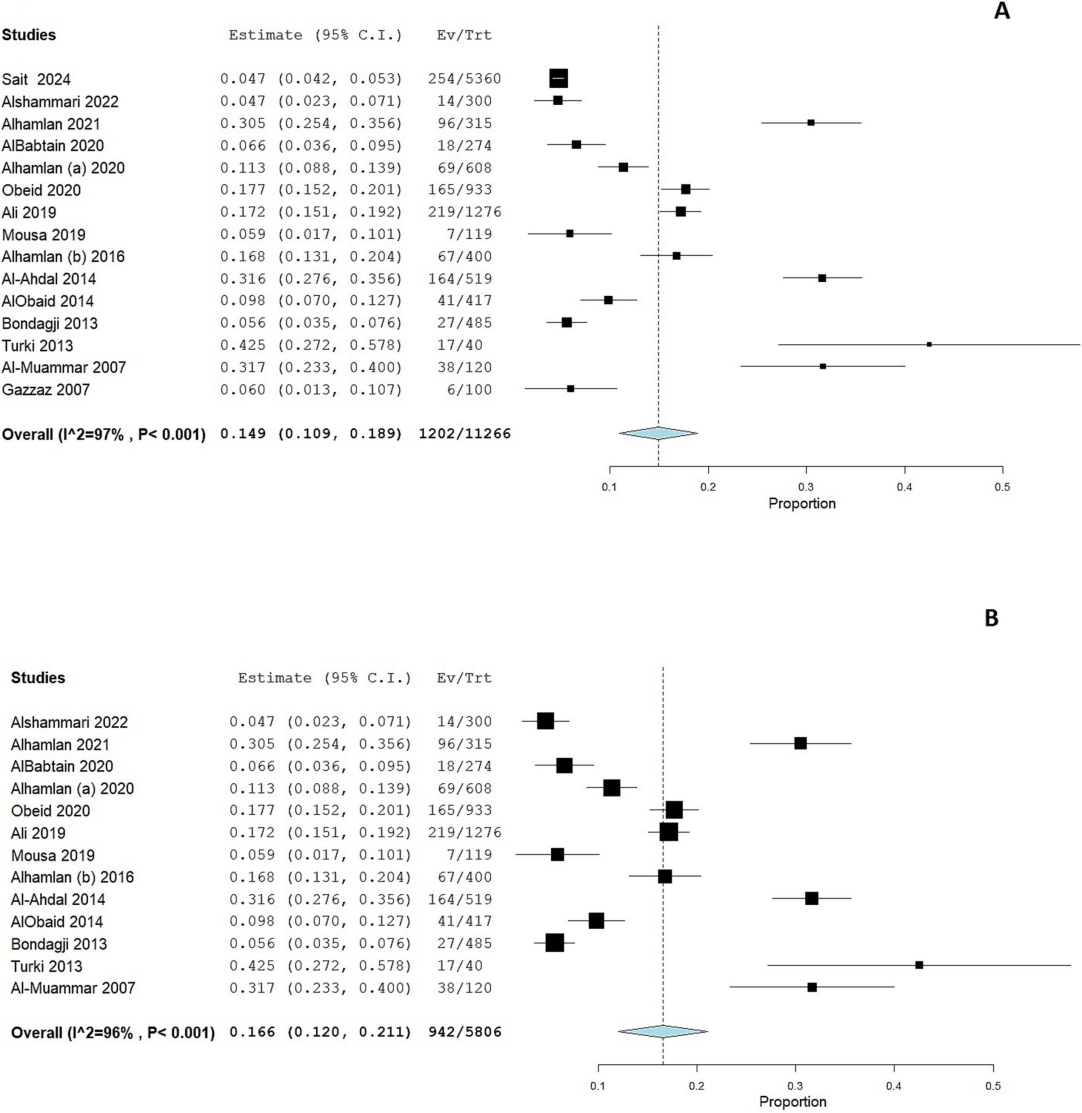
Forest plot of HPV prevalence among women in Saudi Arabia **(A)** and in women who underwent PCR testing **(B)**. CI, confidence interval; PCR, polymerase chain reaction.

A total of 15 studies assessed HPV prevalence among women attending cervical screening, ranging from 4.3 to 43%. The pooled prevalence of HPV infection was 14.9% (95% CI: 10.9 to 18.9%; Figure 2A), with substantial heterogeneity across the included studies (*p* < 0.001; I^2^ = 97.4%). A leave-one-out sensitivity analysis did not resolve this significant heterogeneity. Additionally, when we excluded studies using the HC2 HPV detection method from the pooled analysis, the significant heterogeneity persisted (Pooled prevalence = 16.6% [95% CI: 12 to 21.1%]; I^2^ = 96%; Figure 2B). This reflects substantial differences in the characteristics of the studied populations, methodologies, or regional variations in HPV prevalence.

**Figure 3:**
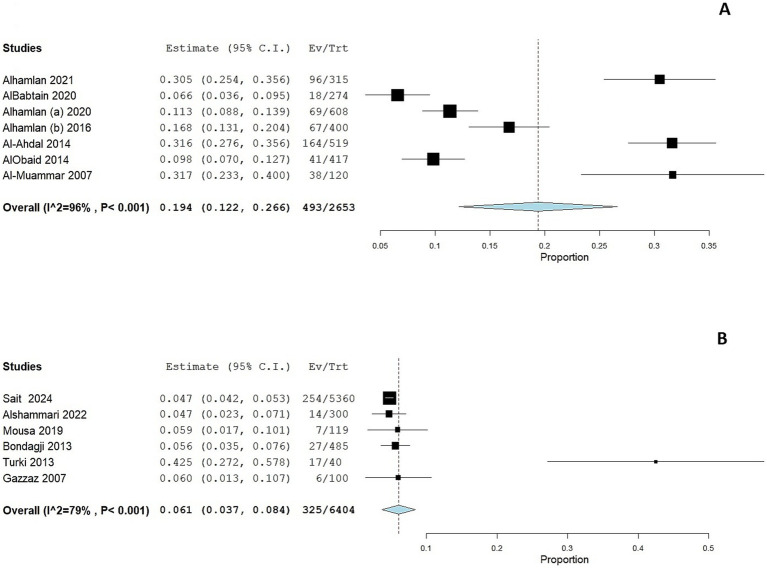
Forest plot of HPV prevalence among women in Saudi Arabia residing in Riyadh **(A)** and in the Western Region **(B)**. CI, confidence interval.

We performed a subgroup analysis according to the geographical location of the study. Eight studies were conducted in Riyadh and showed a pooled prevalence of 19.1% (95% CI: 13.1 to 25%; Figure 3A). However, substantial heterogeneity was present among the included studies (I^2^ = 96%, *p* < 0.001). Six studies were conducted in the Western region of Saudi Arabia, showing a pooled prevalence of 6.1% (95% CI: 3.7 to 8.4%; Figure 3B). However, substantial heterogeneity was present among the included studies (I^2^ = 79%, *p* < 0.001).

We also conducted a subgroup analysis to explore potential temporal trends in HPV prevalence before and after the introduction of the national HPV vaccination program in Saudi Arabia (initiated in 2017). As shown in Supplementary Figure 1, the pooled prevalence of HPV in studies conducted after 2017 (Panel A) was 12.1% (95% CI: 7.1–17.2%; I^2^ = 97.8%), compared to a pooled prevalence of 19.9% (95% CI: 10.2–29.6%; I^2^ = 97.1%) in studies conducted prior to 2017 (Panel B). Although the observed point estimate suggests a lower HPV prevalence in more recent studies, the difference was not statistically significant, and substantial heterogeneity persisted in both subgroups.

A meta-regression analysis was conducted to evaluate whether the mean age of study populations contributed to the heterogeneity in HPV prevalence across studies. There was a non-significant positive association between mean age and HPV prevalence (coefficient = 0.007; 95% CI: −0.004 to 0.018; *p* = 0.2). The omnibus *p*-value for the model was also non-significant (p = 0.2), indicating that age alone did not explain a significant proportion of the between-study variability.

#### 3.3.2 HPV prevalence among women with cytological abnormalities

**Figure 4:**
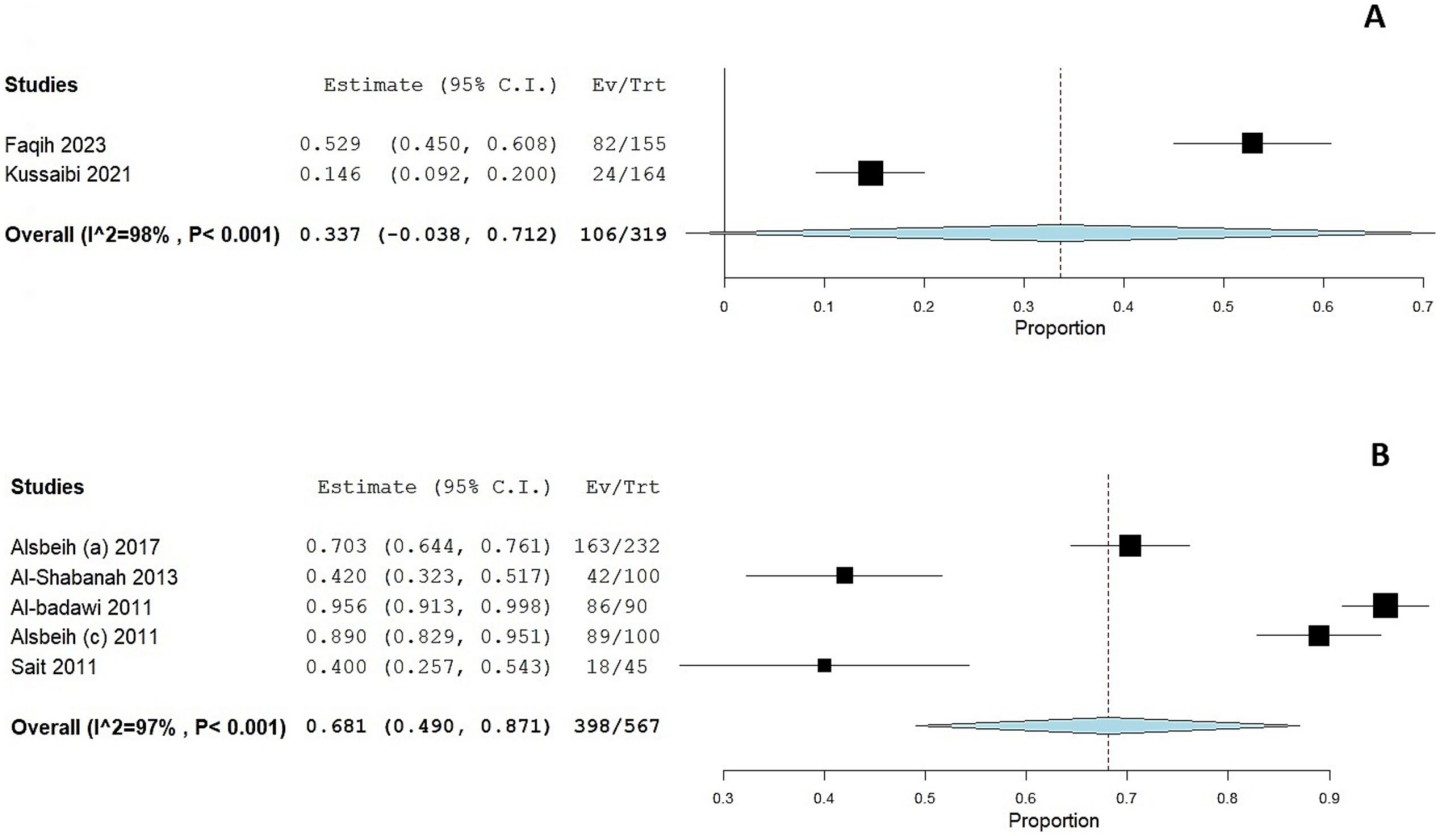
Forest plot of HPV prevalence among women with cytological abnormalities **(A)** and gynecological malignancies **(B)**. CI, confidence interval.

Two studies (30, 33) assessed HPV prevalence among women with cytological abnormalities. The prevalence was considerably higher in these subgroups compared to the general population. The pooled prevalence of HPV infection was 33.7% (95 CI: 0–71.2%; Figure 4A); the pooled prevalence showed substantial heterogeneity (I^2^ = 98%, *p* < 0.001).

#### 3.3.3 HPV prevalence among women with gynecological malignancies

The prevalence of HPV was markedly elevated among women with gynecological malignancies, as reported in five studies (39, 43, 45–47), with a prevalence ranging from 42 to 95.5%. The adjusted pooled prevalence among this cohort was 68.1% (95% CI: 49 to 87.1%; Figure 4B); the pooled prevalence showed substantial heterogeneity (I^2^ = 98%, *p* < 0.001).

#### 3.3.4 Incidence and clearance rate

Only one included study reported the incidence of HPV infection among women residing in Saudi Arabia after a 5-year follow-up period (21). In this study, the reported incidence of HPV infection was 47 per 100,000 person-years. On the other hand, the rate of HPV clearance after 1 year was 84.3%.

### 3.4 HPV genotypes’ distribution

**Figure 5:**
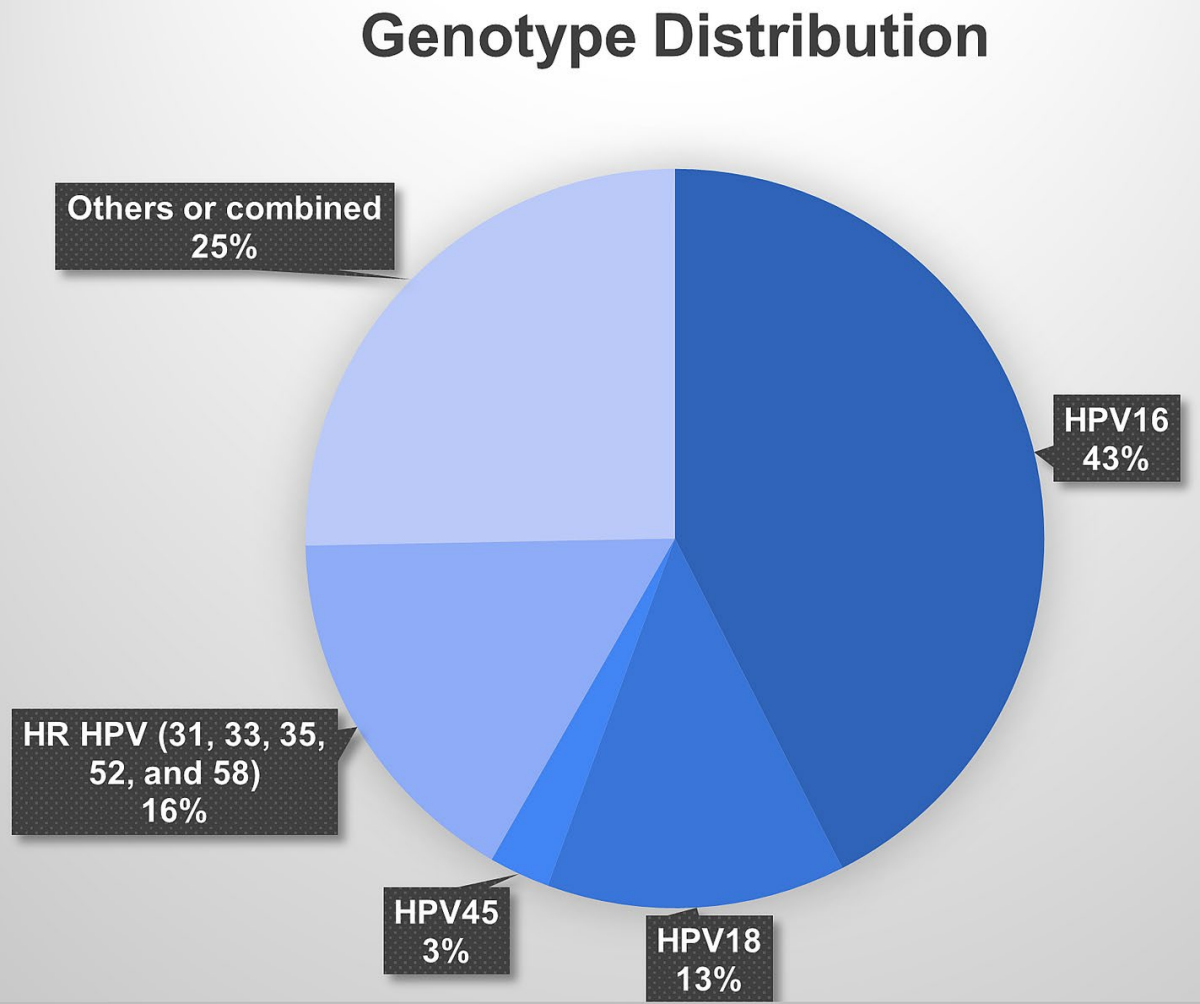
Pie chart of HPV genotypes’ distribution among women in Saudi Arabia.

Among the studies that focused on women attending cervical screening, HPV-16 was the most common genotype, accounting for nearly 35.4% of the reported HPV infections. HPV-18 was also prevalent, accounting for 10.9% of the reported infections. Other high-risk HPV genotypes, such as HPV-45 and types 31, 33, 35, 52, and 58, were less frequently reported, accounting for 2.2 and 13.7% of the reported infections, respectively (Figure 5).

Based on the studies with available data, the pooled prevalence of the HPV-16 genotype among the general population was 4.8% (95% CI: 2.9 to 6.7%; Supplementary Figure 2A), while the pooled prevalence of the HPV-18 genotype was 2.1% (95% CI: 1.1 to 3.1%; Supplementary Figure 2B). The pooled prevalence of the high-risk HPV genotypes (31, 33, 35, 50, 51) and among the general population was 2.1% (95% CI: 0.9 to 3.4%%; Supplementary Figure 3C).

The genotype’s distribution of HPV infection among women with cytological abnormalities or gynecological malignancies was similar to the general population, with HPV-16 and HPV-18 being the most common genotypes (Table 2).

### 3.5 Factors associated with HPV infection

The association of various risk factors with HPV infection among women residing in Saudi Arabia was analyzed across 11 studies, as presented in Table 3. Age was identified as a significant risk factor in multiple studies. For instance, Sait et al. (21) found a statistically significant association between age and HPV infection, with an odds ratio (OR) of 0.98 (95% CI: 0.96–0.99), indicating a slight decrease in risk with increasing age. Conversely, Alshammari et al. (31) reported an OR of 3.01 (95% CI: 1.02–8.88), suggesting increased risk in specific age groups. Parity, higher education, and comorbidities were other significant factors in HPV infection risk in Sait et al. (21). On the other hand, several studies found an association between marital status and HPV infection risk. Alhamlan et al. (32) and Obeid et al. (36) reported significant associations between ever-married status and HPV infection, with *p*-values of 0.003 and 0.002, respectively. Smoking was also identified as a significant risk factor for HPV infection, with an OR of 2.49 (95% CI: 1.40–4.46) (40).

**Table 3:** Risk factors of HPV infection among women residing in Saudi Arabia (*n* = 11 studies).

Study ID	Age	Parity	Higher education	Multiple comorbidities	Diabetes	Hypertension	Ever married	Smoking	Multiple sexual partner	Contraceptive use
Sait et al. 2024 (21)	0.98 (0.96, 0.99)	0.91 (0.86, 0.96)	1.7 (1.1, 2.8)	0.38 (0.20, 0.71)	0.49 (0.30, 0.83)	0.65 (0.43, 0.98)	N/A	N/A	N/A	N/A
Faqih et al. 2023 (30)	HPV-16: (*p*-value = 0.012, coefficient = 5.99); HPV-Others: (p-value = 0.029, coefficient = 3.67)	N/A	N/A	N/A	N/A	N/A	N/A	N/A	N/A	N/A
Alshammari et al. 2022 (31)	3.01 (1.02, 8.88)	N/A	N/A	N/A	N/A	N/A	N/A	N/A	N/A	N/A
Alhamlan et al. 2021 (32)	*p* = 0.013	N/A	N/A	N/A	N/A	N/A	p = 0.003	N/A	N/A	N/A
Kussaibi et al. 2021 (33)	NS	N/A	N/A	N/A	N/A	N/A	N/A	N/A	N/A	N/A
Obeid et al. 2020 (36)	*p* = 0.003	N/A	N/A	N/A	N/A	N/A	*p* = 0.002	N/A	N/A	N/A
Ali et al. 2019 (37)	P < 0.001	N/A	N/A	N/A	N/A	N/A	N/A	N/A	N/A	N/A
Alsbeih et al. 2017 (39)	NS	*p* = 0.049	N/A	N/A	N/A	N/A	N/A	N/A	N/A	N/A
Alhamlan et al. 2016 (40)	NS	NS	NS	N/A	N/A	N/A	NS	2.49 (1.40, 4.46)	3.56 (1.19, 11.3)	NS
AlObaid et al. 2014 (42)	NS	NS	NS	N/A	N/A	N/A	N/A	NS	N/A	N/A
Alsbeih 2011 et al. (46)	*p* = 0.028	N/A	N/A	N/A	N/A	N/A	N/A	N/A	N/A	N/A

Data are presented as odds ratio (95% confidence interval) or *p*-values. N/A, Not available; NS, Not significant.

The presence of multiple sexual partners was significantly associated with HPV infection risk in Alhamlan et al. (40), with an OR of 3.56 (95% CI: 1.19–11.3). The association between COC use and HPV infection was less commonly reported. Among the studies that assessed COC use, none reported a statistically significant association between COC use and HPV infection.

## 4 Discussion

While global trends show a decline in cervical cancer cases due to the success of screening and vaccination programs, Saudi Arabia has seen a 450% rise in cervical cancer incidence since 1990, with an annual mortality of 179 women (23). Despite these alarming figures, the utilization of cervical screening in the region is low, even though routine screening is recommended for women with intraepithelial neoplasia (24). This highlights the urgent need to address the burden of HPV-related diseases, as recent studies predict a significant rise in cervical cancer incidence in Saudi Arabia by 2030 if no further interventions are implemented (23). With the lack of a national registry for HPV infections, there is a need to understand the actual prevalence of HPV infection as a first step toward establishing a comprehensive national screening and vaccination program.

In the present systematic review and meta-analysis, which included 15,224 women across Saudi Arabia, we found that the hospital-based prevalence of HPV infection among the general population residing in Saudi Arabia was 14.9% (95% CI: 10.9 to 18.9%). Such an estimate is comparable to data from the Middle East, where prevalence ranges from 14.7 to 31.3% (37). Our findings also suggest that the prevalence of HPV infections has become comparable to global figures despite historically lower rates in the region. For instance, a previous systematic review found that the global prevalence of HPV infections ranged from 9 to 12% (52). More recent reports also showed that the global prevalence of HPV infections among the general female population ranges from 9.4 to 21.8%, while it was 9–11% among women attending cervical screening (3). Additionally, the current prevalence rates from Saudi Arabia align more closely with those reported in several Western countries, including the United Kingdom [13.2% (53)], Spain [9.6% (50)], and France [11–16% (54)], which may suggest shifting epidemiological patterns in Saudi Arabia. The prevalence in Saudi Arabia also runs in line with estimates from the United Arab Emirates (14.7%) (37), Qatar (8.1%) (55), Oman (17.8%) (56), and Egypt (13.5%) (57). Thus, it is crucial to implement widespread screening and vaccination programs to curb the rising incidence of HPV infections in Saudi Arabia.

Despite the significant burden of HPV in Saudi Arabia, our findings are limited by the substantial variability among the included studies, with a prevalence ranging from 4.3 to 43%. This variability could not be resolved by subgroup analyses according to the geographical location or diagnostic methods, suggesting that other factors, such as differences in study populations, sample sizes, or healthcare access, may contribute to the observed heterogeneity. The wide range of prevalence underscores the need for more standardized, large-scale epidemiological studies to provide a clearer understanding of the actual burden of HPV infection in Saudi Arabia.

Current evidence demonstrates geographical variability in the HPV distribution, with a higher prevalence among developing countries. Previous reports showed higher HPV prevalences in Sub-Saharan Africa, Latin America, Eastern Asia, and Eastern Europe (3). The present systematic review and meta-analysis revealed significant geographical differences in HPV prevalence across Saudi Arabia. The inability of our subgroup analyses to fully explain these regional differences suggests that other underlying factors, such as differences in sexual behaviors, population patterns, and the presence of high-risk subpopulations, might play a role. Moreover, there may be variations in public health initiatives or the availability of HPV screening in different regions, leading to inconsistent reporting of HPV cases. Thus, targeted awareness campaigns, improved access to screening in remote areas, and a better understanding of local risk factors are essential for addressing the disparities in HPV prevalence across different regions in Saudi Arabia.

Several risk factors contribute to the persistence of HPV infection, including high parity, multiple sexual partners, genetic predispositions, smoking, and coinfections with other sexually transmitted infections, and COC use (51, 58). In the present systematic review, several studies from Saudi Arabia have identified important risk factors for HPV infection. Age emerged as a significant factor in multiple studies, as well as parity, higher education, the presence of comorbidities, and smoking (see Table 3). These findings underscore the importance of identifying key demographic, behavioral, and clinical factors contributing to HPV infection risk in Saudi Arabia, as understanding these associations can help inform targeted public health interventions.

There is limited awareness about HPV-related health risks, both in the general population and among individuals who are HPV-positive. In a recent national study from Saudi Arabia, it was found that 88% of Saudi women with cervical cancer did not undergo cervical cancer screening, primarily due to the lack of a physician’s recommendations and lack of knowledge about cervical cancer (23). More notably, nearly 60% of the adults in Saudi Arabia were found to have inadequate knowledge about HPV screening and vaccination (59). Addressing these knowledge gaps is essential for the successful implementation of a national HPV screening and vaccination program.

To our knowledge, this is the first systematic review and meta-analysis that has comprehensively evaluated the prevalence and genotypes’ distribution of HPV infection among women in Saudi Arabia. However, we acknowledge the existence of some limitations. First, there was considerable heterogeneity across the included studies, as indicated by the high I^2^ values, suggesting substantial variability in study designs, populations, and methodologies. We explored potential sources of heterogeneity through meta-regression analyses assessing participants’ age distributions, marital status, and screening uptake; however, these analyses also failed to significantly clarify the observed variability. This persistent heterogeneity suggests that unmeasured or insufficiently reported factors—such as temporal variations, differences in sexual behaviors, socio-economic contexts, or variability in healthcare access—could underlie the observed discrepancies. Thus, our pooled estimates should be interpreted with caution, highlighting the need for standardized, large-scale epidemiological studies to accurately determine HPV prevalence in Saudi Arabia. Second, many of the studies included were based on specific subpopulations, such as women attending tertiary care centers or those with existing gynecological abnormalities, which may not be representative of the general population in Saudi Arabia. Third, there were significant regional differences in the reported prevalence, with some regions, such as Riyadh, having a higher prevalence compared to others, such as Jeddah. This geographic variability may reflect differences in screening practices or population characteristics. Additionally, several studies included in our meta-analysis were retrospective in design and classified as having a high risk of bias, primarily due to potential selection bias, incomplete control of confounding factors, or inadequate reporting standards. Inclusion of such studies might have contributed to biased prevalence estimates, either over or underestimating the actual HPV prevalence. Thus, results should be interpreted with caution. Several included studies lacked comprehensive HPV genotype reporting and were excluded from the genotype-specific meta-analyses. While this approach preserved analytical robustness, it may have introduced reporting bias by omitting potentially informative data. Finally, some studies did not adequately control confounding factors, which are crucial for understanding the actual risk of HPV infection. These limitations emphasize the need for a well-designed national screening survey to provide robust and representative data on HPV prevalence across different regions of Saudi Arabia.

### 4.1 Conclusion

In conclusion, the prevalence of HPV infections among women in Saudi Arabia has become comparable to global figures and recent statistics from Western countries. We demonstrated that the prevalence of HPV infection among women attending cervical screening ranges from 11 to 19%, with a higher prevalence among those with cervical abnormalities or malignancies. The prevalence of high-risk HPV genotypes was notable. Our results also demonstrate that several risk factors predispose to the risk of HPV infection among women residing, including age, parity, education, comorbidities, marital status, smoking, and sexual behavior.

### 4.2 Policy recommendations and future research

Unlike Western nations, Saudi Arabia is still in the early stages of adopting HPV screening and preventive vaccination. The Saudi Ministry of Health recently introduced the HPV vaccine for young schoolgirls aged 9 to 14, but its uptake has been met with resistance due to sociocultural factors. These include a lack of awareness about HPV and its link to cervical cancer and cultural sensitivities around discussing sexually transmitted infections in conservative societies. Additionally, there is a stigma associated with HPV due to its sexual transmission, which can deter individuals and families from seeking vaccination or screening services (60). Since 2017, several countries have fully or partially implemented HPV DNA-based screening (30). As HPV-based screening becomes more prevalent globally, Saudi Arabia will need to consider whether and how to implement similar programs to enhance cervical cancer prevention. However, without accurate data on the actual prevalence of HPV infection in Saudi Arabia, it is difficult to answer whether HPV DNA-based screening should be nationally implemented. Our data highlights significant variability in HPV prevalence within the country.

Thus, as a first step forward, there is a need for a comprehensive, nationwide epidemiological study to assess the actual prevalence of HPV infection and genotype distribution across different regions and demographics. Additionally, a gradual transition from Pap smear-based screening to HPV DNA-based testing, following the successful example of other countries, would allow for earlier detection of high-risk HPV types, enabling more timely interventions and reducing cervical cancer rates. As a long-term strategy, there is a need to strengthen the implementation of HPV vaccination programs and establish a national registry to track HPV infection rates and vaccination coverage.

## Data Availability

The original contributions presented in the study are included in the article/Supplementary material, further inquiries can be directed to the corresponding author.

## Footnotes

^1^ https://new.rayyan.ai/

## References

[1] Chesson HW Dunne EF Hariri S Markowitz LE. The estimated lifetime probability of acquiring human papillomavirus in the United States. Sex Transm Dis. (2014) 41:660–4. doi: 10.1097/OLQ.0000000000000193, PMID: 25299412 PMC6745688

[2] Bruni L Albero G Serrano B Mena M Gómez D Muñoz J . Human papillomavirus and related diseases in Barbados. Summary report 17 June 2019. ICO/IARC Inf Cent HPVand Cancer (HPV Inf Centre). (2019). Available at: https://hpvcentre.net/statistics/reports/BRB.pdf

[3] Kombe Kombe AJ Li B Zahid A Mengist HM Bounda GA Zhou Y . Epidemiology and burden of human papillomavirus and related diseases, molecular pathogenesis, and vaccine evaluation. Front Public Health. (2021) 8:552028. doi: 10.3389/fpubh.2020.552028, PMID: 33553082 PMC7855977

[4] Lekoane KMB Kuupiel D Mashamba-Thompson TP Ginindza TG. Evidence on the prevalence, incidence, mortality and trends of human papilloma virus-associated cancers in sub-Saharan Africa: systematic scoping review. BMC Cancer. (2019) 19:563. doi: 10.1186/s12885-019-5781-3, PMID: 31185951 PMC6558783

[5] Lewis RM Laprise JF Gargano JW Unger ER Querec TD Chesson HW . Estimated prevalence and incidence of disease-associated human papillomavirus types among 15- to 59-year-olds in the United States. Sex Transm Dis. (2021) 48:273–7. doi: 10.1097/OLQ.0000000000001356, PMID: 33492097 PMC10037549

[6] Petca A Borislavschi A Zvanca M Petca R-C Sandru F Dumitrascu M. Non-sexual HPV transmission and role of vaccination for a better future (review). Exp Ther Med. (2020) 20:1. doi: 10.3892/etm.2020.9316, PMID: 33101476 PMC7579832

[7] Kero K Rautava J. HPV infections in heterosexual couples: mechanisms and covariates of virus transmission. Acta Cytol. (2019) 63:143–7. doi: 10.1159/000494710, PMID: 30799413

[8] Abdulsalam EA Nofal A El-Ghareeb MI. Role of HPV vaccines in multiple recalcitrant warts treatment: review article. Egypt J Hosp Med. (2022) 87:1173–6. doi: 10.21608/ejhm.2022.223154

[9] Walboomers JMM Jacobs MV Manos MM Bosch FX Kummer JA Shah KV . Human papillomavirus is a necessary cause of invasive cervical cancer worldwide. J Pathol. (1999) 189:12–9. 10451482 10.1002/(SICI)1096-9896(199909)189:1<12::AID-PATH431>3.0.CO;2-F

[10] Markowitz LE Schiller JT. Human Papillomavirus Vaccines. J Infect Dis. (2021) 224:S367–78. doi: 10.1093/infdis/jiaa621, PMID: 34590141 PMC8577198

[11] Gargano J Meites E Watson M Unger E Markowitz L. Human Papillomavirus In: VPD surveillance manual eds. Roush and Laura Baldy. Atlanta, Georgia: Centers for Disease Control and Prevention (CDC), based. (2017). 1–15.

[12] Park E Kim JY Choi S Kim DS Oh YL. Carcinogenic risk of human papillomavirus (HPV) genotypes and potential effects of HPV vaccines in Korea. Sci Rep. (2019) 9:12556. doi: 10.1038/s41598-019-49060-w, PMID: 31467383 PMC6715722

[13] Huber J Mueller A Sailer M Regidor PA. Human papillomavirus persistence or clearance after infection in reproductive age. What is the status? Review of the literature and new data of a vaginal gel containing silicate dioxide, citric acid, and selenite. Women Health. (2021) 17:17455065211020702. doi: 10.1177/17455065211020702, PMID: 34096424 PMC8785287

[14] Graham SV. The human papillomavirus replication cycle, and its links to cancer progression: a comprehensive review. Clin Sci. (2017) 131:2201–21. doi: 10.1042/CS20160786, PMID: 28798073

[15] Dabán-López P Fernández-Martínez NF Petrova D Rodríguez-Barranco M Jiménez-Moleón JJ Gutierrez J . Epidemiology of human papillomavirus-associated anogenital cancers in Granada: a three-decade population-based study. Front Public Health. (2023) 11:1205170. doi: 10.3389/fpubh.2023.1205170, PMID: 37780447 PMC10537955

[16] Whitton AF Knight GL Marsh EK. Risk factors associated with oral human papillomavirus (HPV) prevalence within a young adult population. BMC Public Health. (2024) 24:1485. doi: 10.1186/s12889-024-18977-x, PMID: 38831431 PMC11145846

[17] Bovo AC Pedrão PG Guimarães YM Godoy LR Resende JCP Longatto-Filho A . Combined Oral contraceptive use and the risk of cervical Cancer: literature review. Rev Bras Ginecol e Obstet. (2023) 45:E818–24. doi: 10.1055/s-0043-1776403, PMID: 38141603 PMC10748515

[18] Mahfouz MS Elmahdy M Ryani MA Abdelmola AO Kariri SAA Alhazmi HYA . Contraceptive use and the associated factors among women of reproductive age in Jazan City, Saudi Arabia: a cross-sectional survey. Int J Environ Res Public Health. (2023) 20:843. doi: 10.3390/ijerph20010843, PMID: 36613165 PMC9820157

[19] Schreuder A Mokadem I Smeets NJL Spaanderman MEA Roeleveld N Lupattelli A . Associations of periconceptional oral contraceptive use with pregnancy complications and adverse birth outcomes. Int J Epidemiol. (2023) 52:1388–99. doi: 10.1093/IJE/DYAD045, PMID: 37040615 PMC10555752

[20] Brisson M Kim JJ Canfell K Drolet M Gingras G Burger EA . Impact of HPV vaccination and cervical screening on cervical cancer elimination: a comparative modelling analysis in 78 low-income and lower-middle-income countries. Lancet. (2020) 395:575–90. doi: 10.1016/S0140-6736(20)30068-4, PMID: 32007141 PMC7043009

[21] Sait KH Anfinan NM Sait HK Basalamah HA. Human papillomavirus prevalence and dynamics: insights from a 5-year population-based study in Jeddah, Kingdom of Saudi Arabia. Saudi Med J. (2024) 45:252–60. doi: 10.15537/smj.2024.45.3.20230824, PMID: 38438209 PMC11115394

[22] Turki R Sait K Anfinan N Sohrab SS Abuzenadah AM. Prevalence of human papillomavirus in women from Saudi Arabia. Asian Pac J Cancer Prev. (2013) 14:3177–81. doi: 10.7314/APJCP.2013.14.5.3177 23803100

[23] Alkhamis FH Alabbas ZAS Al Mulhim JE Alabdulmohsin FF Alshaqaqiq MH Alali EA. Prevalence and predictive factors of cervical Cancer screening in Saudi Arabia: a Nationwide study. Cureus. (2023) 15:e49331. doi: 10.7759/cureus.49331, PMID: 38143659 PMC10748848

[24] Mahmoud Al-Mandeel H Sagr E Sait K Latifah HM Al-Obaid A Al-Badawi IA . Clinical practice guidelines on the screening and treatment of precancerous lesions for cervical cancer prevention in Saudi Arabia. Ann Saudi Med. (2016) 36:313–20. doi: 10.5144/0256-4947.2016.313, PMID: 27710981 PMC6074318

[25] Farahat FM Faqih NT Alharbi RS Mudarris RI Alshaikh SA Al-Jifree HM. Epidemiological characteristics of cervical cancer in a tertiary care hospital, western Saudi Arabia. Saudi Med J. (2021) 42:338–41. doi: 10.15537/smj.2021.42.3.20200603 33632914 PMC7989268

[26] Page MJ McKenzie JE Bossuyt PM Boutron I Hoffmann TC Mulrow CD . The PRISMA 2020 statement: an updated guideline for reporting systematic reviews. BMJ. (2021) 372:n71. doi: 10.1136/BMJ.N71, PMID: 33782057 PMC8005924

[27] Herzog R Álvarez-Pasquin MJ Díaz C Del Barrio JL Estrada JM Gil Á. Are healthcare workers intentions to vaccinate related to their knowledge, beliefs and attitudes? A systematic review. BMC Public Health. (2013) 13. doi: 10.1186/1471-2458-13-154, PMID: 23421987 PMC3602084

[28] Wells G Shea B O’Connell D Peterson J, The Newcastle-Ottawa scale (NOS) for assessing the quality of nonrandomised studies in meta-analyses. Ottawa Hosp Res Inst (2021). Available online at: http://www.ohri.ca/programs/clinical_epidemiology/oxford.asp [Accessed February 12, 2022]

[29] Wallace BC Schmid CH Lau J Trikalinos TA. Meta-analyst: software for meta-analysis of binary, continuous and diagnostic data. BMC Med Res Methodol. (2009) 9:80. doi: 10.1186/1471-2288-9-80, PMID: 19961608 PMC2795760

[30] Faqih L Alzamil L Aldawood E Alharbi S Muzzaffar M Moqnas A . Prevalence of human papillomavirus infection and cervical abnormalities among women attending a tertiary Care Center in Saudi Arabia over 2 years. Trop Med Infect Dis. (2023) 8:511. doi: 10.3390/tropicalmed8120511, PMID: 38133443 PMC10747865

[31] Alshammari FD Alharbi SA Humaida MI Abdalhabib EK Bealy MAB Elkhalifa AEO . Human papillomavirus genotypes associated with cervical intraepithelial lesions among Saudi women. Eur Rev Med Pharmacol Sci. (2022) 26:6367–73. doi: 10.26355/eurrev_202209_29663, PMID: 36111939

[32] Alhamlan F Obeid D Khayat H Asma T Al-Badawi IA Almutairi A . Prognostic impact of human papillomavirus infection on cervical dysplasia, cancer, and patient survival in Saudi Arabia: a 10-year retrospective analysis. Ann Saudi Med. (2021) 41:350–60. doi: 10.5144/0256-4947.2021.350, PMID: 34873934 PMC8650596

[33] Kussaibi H Al Dossary R Ahmed A Muammar A Aljohani R. Correlation of high-risk HPV genotypes with pap test findings: a retrospective study in Eastern Province. Saudi Arabia Acta Cytol. (2021) 65:48–55. doi: 10.1159/000509669, PMID: 32784299

[34] AlBabtain FA Hussain AN Alsoghayer SA Alwahbi OA Almohaisen N Alkhenizan AH. The yield of pap smears and its characteristics in a community based setting in Saudi Arabia. Saudi Med J. (2020) 41:661–5. doi: 10.15537/SMJ.2020.6.25085, PMID: 32518936 PMC7502935

[35] Alhamlan FS Khayat HH Obeid DA Tulba AM Baduwais TS Alfageeh MB . Clinical comparison of two human papillomavirus detection assays: GenoFlow and reverse line blot. J Infect Dev Ctries. (2020) 14:97–103. doi: 10.3855/jidc.11769, PMID: 32088690

[36] Obeid DA Almatrrouk SA Khayat HH Al-Muammer TA Tulbah AM Albadawi IA . Human papillomavirus type 16 and 18 viral loads as predictors associated with abnormal cervical cytology among women in Saudi Arabia. Heliyon. (2020) 6:e03473. doi: 10.1016/j.heliyon.2020.e03473, PMID: 32140590 PMC7047185

[37] Ali MAM Bedair RN Abd El Atti RM. Cervical high-risk human papillomavirus infection among women residing in the Gulf cooperation council countries: prevalence, type-specific distribution, and correlation with cervical cytology. Cancer Cytopathol. (2019) 127:567–77. doi: 10.1002/cncy.22165, PMID: 31390155

[38] Mousa M Al-Amri SS Degnah AA Tolah AM Abduljabbar HH Oraif AM . Prevalence of human papillomavirus in Jeddah. Saudi Arabia Ann Saudi Med. (2019) 39:403–9. doi: 10.5144/0256-4947.2019.403, PMID: 31804132 PMC6894452

[39] Alsbeih GA Al-Harbi NM Bin Judia SS Khoja HA Shoukri MM Tulbah AM. Reduced rate of human papillomavirus infection and genetic overtransmission of TP53 72C polymorphic variant lower cervical cancer incidence. Cancer. (2017) 123:2459–66. doi: 10.1002/cncr.30635, PMID: 28393355 PMC5485004

[40] Alhamlan FS Khayat HH Ramisetty-Mikler S Al-Muammar TA Tulbah AM Al-Badawi IA . Sociodemographic characteristics and sexual behavior as risk factors for human papillomavirus infection in Saudi Arabia. Int J Infect Dis. (2016) 46:94–9. doi: 10.1016/j.ijid.2016.04.004, PMID: 27062984

[41] Al-Ahdal MN Al-Arnous WK Bohol MFF Abuzaid SM Shoukri MM Elrady KS . Human papillomaviruses in cervical specimens of women residing in Riyadh, Saudi Arabia: a hospital-based study. J Infect Dev Ctries. (2014) 8:320–5. doi: 10.3855/jidc.4220 24619263

[42] AlObaid A Al-Badawi IA Al-Kadri H Gopala K Kandeil W Quint W . Human papillomavirus prevalence and type distribution among women attending routine gynecological examinations in Saudi Arabia. BMC Infect Dis. (2014) 14:643–8. doi: 10.1186/s12879-014-0643-8, PMID: 25496614 PMC4272558

[43] Al-Shabanah OA Hafez MM Hassan ZK Sayed-Ahmed MM Abozeed WN Al-Rejaie SS . Human papillomavirus genotyping and integration in ovarian cancer Saudi patients. Virol J. (2013) 10:1–9. doi: 10.1186/1743-422X-10-343, PMID: 24252426 PMC3842654

[44] Bondagji NS Gazzaz FS Sait K Abdullah L. Prevalence of high-risk human papillomavirus infections in healthy Saudi women attending gynecologic clinics in the western region of Saudi Arabia. Ann Saudi Med. (2013) 33:13–7. doi: 10.5144/0256-4947.2013.13, PMID: 23458934 PMC6078578

[45] Al-Badawi IA Al-Suwaine A Al-Aker M Asaad L Alaidan A Tulbah A . Detection and genotyping of human papilloma virus in cervical cancer specimens from Saudi patients. Int J Gynecol Cancer. (2011) 21:907–10. doi: 10.1097/IGC.0b013e318214219f, PMID: 21697680

[46] Alsbeih G Ahmed R Al-Harbi N Venturina LA Tulbah A Balaraj K. Prevalence and genotypes’ distribution of human papillomavirus in invasive cervical cancer in Saudi Arabia. Gynecol Oncol. (2011) 121:522–6. doi: 10.1016/j.ygyno.2011.01.033, PMID: 21353296

[47] Gazzaz SK. Molecular tests to detect human papillomavirus infection in patients with cervical dysplasia and invasive cervical cancer in Saudi Arabia. Med Int Pathol Lab. (2011) 25:3059. doi: 10.2147/plmi.s23059, PMID: 40296898

[48] Al-Muammar T Al-Ahdal MN Hassan A Kessie G Dela Cruz DM Mohamed GE. Human papilloma virus-16/18 cervical infection among women attending a family medical clinic in Riyadh. Ann Saudi Med. (2007) 27:1–5. doi: 10.5144/0256-4947.2007.1, PMID: 17277496 PMC6077029

[49] Gazzaz FSB. Molecular testing of human papillomavirus in cervical specimens. Saudi Med J. (2007) 28:1810–8. PMID: 18060207

[50] Garcia S Dominguez-Gil M Gayete J Rojo S Muñoz JL Salas JS . Prevalence of human papillomavirus in Spanish women from a population screening program. Rev Esp Quimioter. (2017) 30:177–82. PMID: 28508620

[51] Dahlström LA Andersson K Luostarinen T Thoresen S Ögmundsdottír H Tryggvadottír L . Prospective seroepidemiologic study of human papillomavirus and other risk factors in cervical cancer. Cancer Epidemiol Biomarkers Prev. (2011) 20:2541–50. doi: 10.1158/1055-9965.EPI-11-0761, PMID: 21994401

[52] Bruni L Diaz M Castellsagué X Ferrer E Bosch FX De Sanjosé S. Cervical human papillomavirus prevalence in 5 continents: Meta-analysis of 1 million women with normal cytological findings. J Infect Dis. (2010) 202:1789–99. doi: 10.1086/657321, PMID: 21067372

[53] Anderson L O’Rorke M Jamison J Wilson R Gavin A. Prevalence of human papillomavirus in women attending cervical screening in the UK and Ireland: new data from northern Ireland and a systematic review and meta-analysis. J Med Virol. (2013) 85:295–308. doi: 10.1002/jmv.23459 23161367

[54] Monsonego J Zerat L Syrjänen K Zerat JC Smith JS Halfon P. Prevalence of type-specific human papillomavirus infection among women in France: implications for screening, vaccination, and a future generation of multivalent HPV vaccines. Vaccine. (2012) 30:5215–21. doi: 10.1016/j.vaccine.2012.06.013, PMID: 22713720

[55] Elmi AA Bansal D Acharya A Skariah S Dargham SR Abu-Raddad LJ . Human papillomavirus (HPV) infection: molecular epidemiology, genotyping, seroprevalence and associated risk factors among Arab women in Qatar. PLoS One. (2017) 12:e0169197. doi: 10.1371/journal.pone.0169197, PMID: 28046025 PMC5207789

[56] Al-Lawati Z Khamis FA Al-Hamdani A Al-Kalbani M Ramadhan FA Al-Rawahi TR . Prevalence of human papilloma virus in Oman: genotypes 82 and 68 are dominating. Int J Infect Dis. (2020) 93:22–7. doi: 10.1016/j.ijid.2019.12.038, PMID: 31935539

[57] Ashry M Shawky S Mounir Z Fathy F Elsayed H Kamal W . Prevalence and risk factors of human papilloma virus infection among women living with HIV, Egypt, a cross sectional study. BMC Public Health. (2024) 24:1821. doi: 10.1186/s12889-024-19240-z, PMID: 38978047 PMC11232173

[58] Jensen KE Schmiedel S Norrild B Frederiksen K Iftner T Kjaer SK. Parity as a cofactor for high-grade cervical disease among women with persistent human papillomavirus infection: a 13-year follow-up. Br J Cancer. (2013) 108:234–9. doi: 10.1038/bjc.2012.513, PMID: 23169283 PMC3553518

[59] Turki YM Alqurashi J. Knowledge, attitudes, and perceptions towards human papillomavirus (HPV) vaccination among adult women in primary health care centers in Makkah. Saudi Arabia Cureus. (2023) 15:e44157. doi: 10.7759/cureus.44157, PMID: 37638260 PMC10460136

[60] Alhusayn KO Alkhenizan A Abdulkarim A Sultana H Alsulaiman T Alendijani Y. Attitude and hesitancy of human papillomavirus vaccine among Saudi parents. J Family Med Prim Care. (2022) 11:2909–16. doi: 10.4103/jfmpc.jfmpc_2377_21, PMID: 36119278 PMC9480641

