# Supplementary material for: Prevalence and genotypes’ distribution of human papillomavirus among women in Saudi Arabia: a systematic review and meta-analysis
Source: Front Public Health. 2025 May 15;13:1580699. doi: 10.3389/fpubh.2025.1580699 (PMC12119489; doi:10.3389/fpubh.2025.1580699)
Supplement: Supplementary file 1 [file Data_Sheet.DOCX]

Supplementary Material

## Supplementary Figures

**
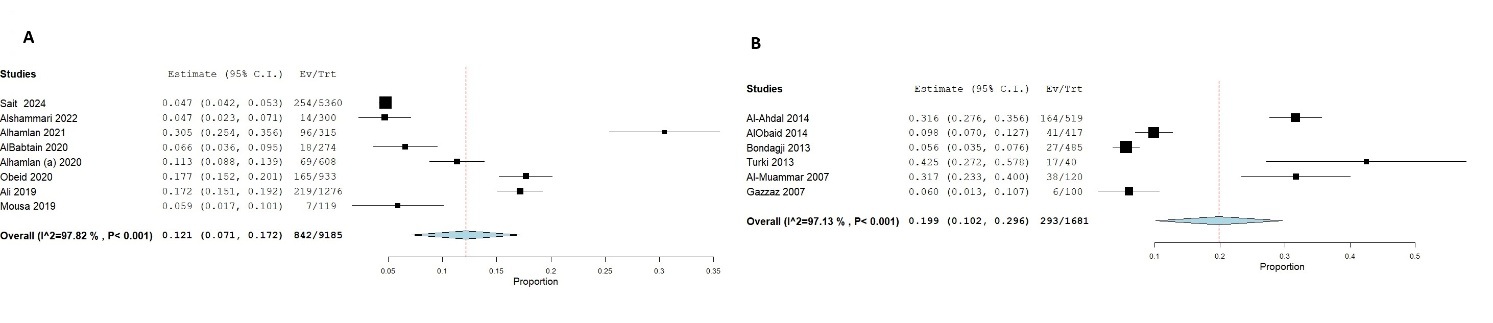
**

**Supplementary Figure 2.** Forest plots showing subgroup analysis of HPV prevalence after (a) and before (b) the introduction of HPV vaccination in Saudi Arabia in 2017. CI: Confidence Interval.

**
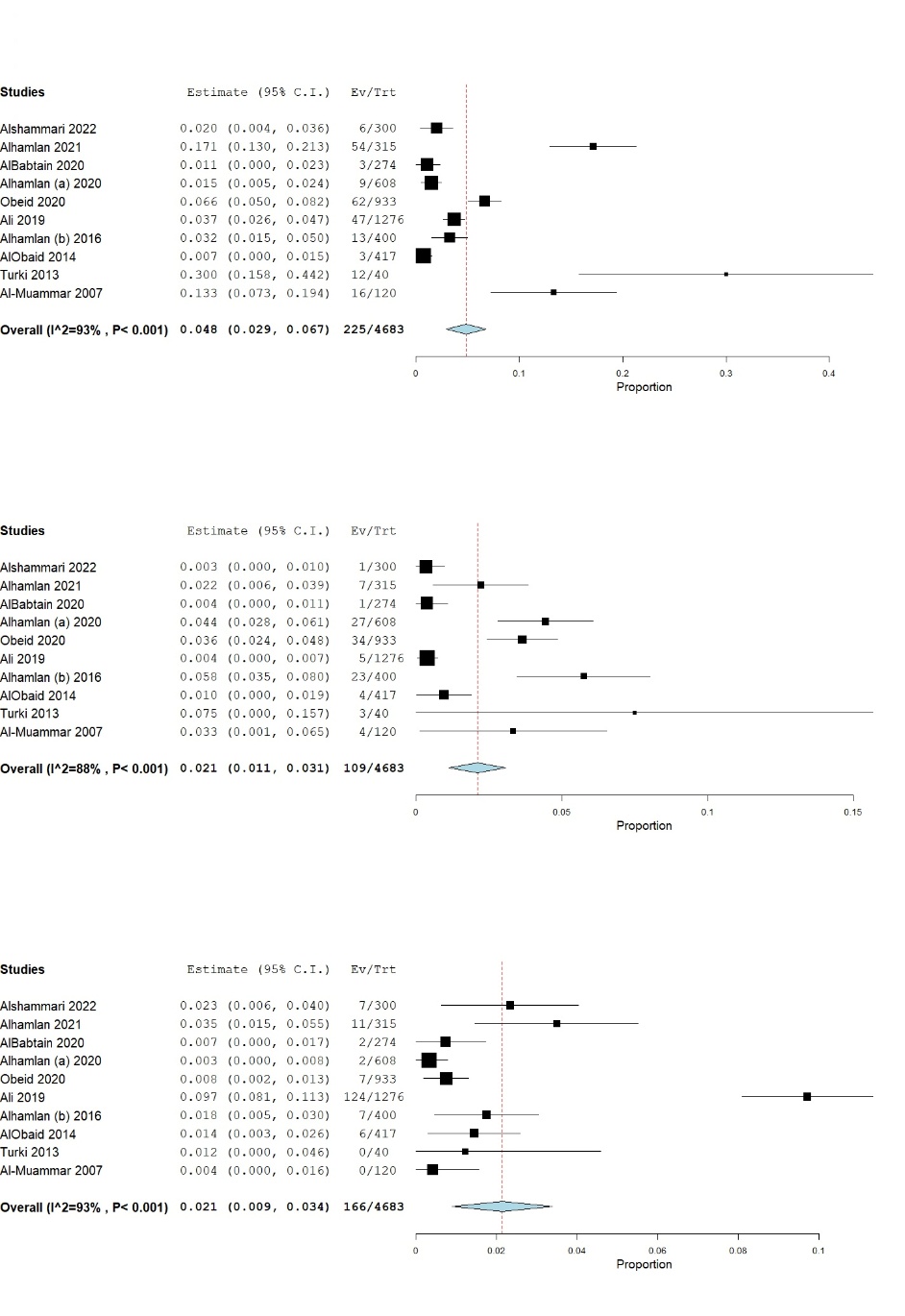
**

**Supplementary Figure 2.** Forest Plot of HPV-16 genotype (a), HPV-18 genotype (b), and high-risk HPV genotypes (c). CI: Confidence Interval.

## Supplementary Tables

Table 1: Search Strategy and Keywords

| **Database** | **Search strategy** | **No. of retrieved citations** |
| --- | --- | --- |
| **PubMed** | #1: ("Papillomavirus Infections"[Mesh] OR "Human papillomavirus 16"[Mesh] OR "Human papillomavirus 18"[Mesh] OR "Papillomaviridae"[Mesh] OR  "Human Papillomavirus"[tiab] OR "HPV"[tiab] OR "Papillomavirus"[tiab] OR "HPV infection"[tiab] OR "Genital warts"[tiab] OR "HPV 16"[tiab] OR "HPV 18"[tiab]) | 80.975 |
|  | #2: ("Saudi Arabia"[Mesh] OR "Kingdom of Saudi Arabia"[tiab] OR "KSA"[tiab] OR "Gulf Region"[tiab] OR "Middle East"[tiab])) | 39,101 |
|  | #3: (1 AND 2) | 132 |
| **Scopus** | TITLE-ABS-KEY("Human Papillomavirus" OR "HPV" OR "Papillomavirus" OR "HPV infection" OR "Papillomavirus infection" OR "Genital warts" OR "HPV 16" OR "HPV 18" OR "High-risk HPV" OR "Low-risk HPV" OR "HPV genotypes" OR "HPV variants" OR "HPV strains") AND TITLE-ABS-KEY("Saudi Arabia" OR "Arab women" OR "Women in Saudi Arabia" OR "Middle Eastern women" OR "Gulf countries" OR "Women in Gulf countries") | 136 |
| **Web of Science: Core databases** | ("Human Papillomavirus" OR "HPV" OR "Papillomavirus" OR "HPV infection" OR "Papillomavirus infection" OR "Genital warts" OR "HPV 16" OR "HPV 18" OR "High-risk HPV" OR "Low-risk HPV" OR "HPV genotypes" OR "HPV variants" OR "HPV strains") AND ("Saudi Arabia" OR "Arab women" OR "Women in Saudi Arabia" OR "Middle Eastern women" OR "Gulf countries" OR "Women in Gulf countries") | 474 |
| **OVID** | ((human papillomavirus OR 'hpv infection' OR 'papillomavirus infection' OR 'genital warts' OR 'hpv 16' OR 'hpv 18' OR 'high-risk hpv' OR 'low-risk hpv' OR 'hpv genotypes' OR 'hpv variants' OR 'hpv strains').ti,ab,kw.)  AND  ((saudi arabia OR 'arab women' OR 'middle eastern women' OR 'women in saudi arabia' OR 'gulf countries' OR 'women in gulf countries').ti,ab,kw.) | 15 |
| **CINAHL** | (TI "Human Papillomavirus" OR TI "HPV" OR TI "Papillomavirus" OR TI "HPV infection" OR TI "Papillomavirus infection" OR TI "Genital warts" OR TI "HPV 16" OR TI "HPV 18" OR AB "Human Papillomavirus" OR AB "HPV" OR AB "Papillomavirus" OR AB "HPV infection" OR AB "Papillomavirus infection" OR AB "Genital warts" OR AB "HPV 16" OR AB "HPV 18" OR MW "Human Papillomavirus" OR MW "HPV" OR MW "Papillomavirus" OR MW "HPV infection" OR MW "Genital warts" OR MW "HPV 16" OR MW "HPV 18") AND (TI "Saudi Arabia" OR TI "Arab women" OR TI "Women in Saudi Arabia" OR TI "Middle Eastern women" OR AB "Saudi Arabia" OR AB "Arab women" OR AB "Women in Saudi Arabia" OR AB "Middle Eastern women" OR MW "Saudi Arabia" OR MW "Arab women" OR MW "Women in Saudi Arabia" OR MW "Middle Eastern women") | 31 |

**Table 2: Baseline characteristics of the included studies (n =22 studies)**

| **Study ID** | **Age in years, mean (SD)** | **Saudi nationality, no (%)** | **No formal education, no (%)** | **Maital status, no (%)** | | | | **At least one comorbidity, no (%)** | **COCs use, no (%)** | **Number of pregnancies, median (range)** | **Smoking, no (%)** | **Histopathological distribution of analyzed cases for HPV genotyping** |
| --- | --- | --- | --- | --- | --- | --- | --- | --- | --- | --- | --- | --- |
|  |  |  |  | Never married | Married | Divorced | Widowed |  |  |  |  |  |
| Sait et al. 2024 (1) | 44.3 (8.3) | NA | 286 (5.3%) | 0 | 4956 (92.5%) | 250 (4.7%) | 154 (2.9%) | 1504 (28.1%) | 1688 (31.5%) | NA | 432 (8.1%) | NA |
| Faqih et al. 2023 (2) | 20-29 Y: 21 (13.6%) 30-39 Y: 76 (49%) 40-49 Y: 43 (27.7%) 50-59 Y: 11 (7.1%) > 60 Y: 4 (2.6%) | 137 (88.4%) | NA | NA | | | | N/A | NA | NA | NA | ASCUS 83 (53.5%) LSILs 46 (29.70%) HSILs 14 (9%) ASC-H 10 (6.50%) SCC 2 (1.3%) |
| Alshammari et al. 2022 (3) | 20-30 Y: 32 (10.6%)  31-40 Y: 83 (27.6%)  41-50 Y: 80 (26.6%)  51-60 Y: 73 (24.3%)  > 60 Y: 32 (10.6%) | 300 (100%) | NA | NA | | | | N/A | NA | NA | NA | Mild atypia 10 (3.3%) Medium Atypia 11 (3.6%) Severe atypia 12 (4%) Normal 267 (89%) |
| Alhamlan et al. 2021 (4) | 49.7 (13.3) | 272 (86.34%) | NA | 31 (9.8%) | 235 (74.6%) | 12 (3.05%) | 37 (11.7%) | N/A | NA | NA | NA | Normal 82 (25.7%) CIN I 54 (16.9%) CIN II 16 (5.01%) CIN III 45 (14.10%) Cervical cancer 118 (36.9%) |
| Kussaibi et al. 2021 (5) | 21–30 Y: 26 (15.9%) 31–40 Y: 65 (39.6%) 41–50 Y: 43 (26.2%) 51–60 Y: 24 (14.6%) >60 Y: 6 ( 3.7%) | 164 (100%) | NA | NA | | | | N/A | NA | NA | NA | Negative 129 (78.7%) ASCUS 243 (14.6%) LSIL 8 (4.9%) AGC 3 (1.8%) |
| AlBabtain 2020 (6) | 47 (11.9) | 1840 (55%) | NA | NA | | | | N/A | NA | 3.5 (2.4)* | NA | Squamous cells 69 (2.1%) Atypical squamous cells of undetermined significance 48 (1.4%) Atypical squamous cells, cannot exclude high-grade lesions 3 (0.1%) Low-grade squamous intraepithelial lesions 16 (0.5%) High-grade squamous intraepithelial lesions 2 (0.1%) Glandular cells 5 (0.1%) Atypical glandular cells 3 (0.1%) Adenocarcinoma 2 (0.1%) |
| Alhamlan 2020 (7) | 11–30 Y: 95 (15.63%) 31–45 Y: 264 (43.42%) 46–60 Y: 209 (34.38%) >60 Y: 40 (6.58%) | 486 (80.07%) | NA | 45 (7.40%) | 530 (87.17%) | 16 (2.6%) | 15 (2.46%) | N/A | NA | NA | NA | NIEL 552 (93.40%) ASCUS 18 (3.05%) LGSIL 14 (2.37%) HGSIL 4 (0.68%) Cervical cancer 3 (0.51%) Unknown 17 (2.79%) |
| Obeid 2020 (8) | 45 (range, 11–95) | 759 (81.4%) | NA | NA | | | | N/A | NA | NA | NA | Normal 635 (68.1%) ASCUS 18 (1.9%) LGSIL 68 (7.2%) HGSIL 65 (6.9%) Cervical Cancer 121 (12.9%) UNK 26 (0.5%) |
| Ali 2019 (9) | NA | NA | NA | NA | | | |  | NA | NA | NA | N/A |
| Mousa 2019 (10) | 31.8 (6.5) | 100 (84%) | NA | 0 | 119 (100%) | 0 | 0 | N/A | NA | NA | NA | NA |
| Alsbeih 2017 (11) | 46 (range 29-78)^#^ | NA | NA | NA |  |  |  | 94 (41%) | NA | 7 (0-16) | NA | AC: 31 (19%) SCC: 132 (81%) Stage I: 42 (26%) Stage II: 78 (48%) Stage III: 36 (22%) Stage IV: 7 (4%) |
| Alhamlan 2016 (12) | 41.2 (10.4) | 291 (73%) | 0 | 0 | 350 (87.5%) | 38 (9.5%) | 12 (12%) | N/A | 287 (72%) | NA | 119 (12.5%) | NA |
| Al-Ahdal 2014 (13) | 37 (9) | NA | NA | NA | | | | N/A | NA | NA | NA | NA |
| AlObaid 2014 (14) | 41.9 (10.45) | 321 (77%) | 21 (6.58%) | NA | | | |  | NA | 0: 8 (2.5%) 1-2: 100 (31.34%) 3-5: 112 (35.01%) | No 273 (85.57%) Yes 44 (13.79%) | NA |
| Al-Shabanah 2013 (15) | 50 (11) | NA | NA | NA | | | | N/A | NA | NA | NA | NA |
| Bondagji 2013 (16) | 44.7 (range 19 - 91) | 485 (100%) | NA | 0 | 470 (86%) | 44 (9%) | 24 (5%) | N/A | NA | 3.5 (range 1-9)* | NA | NA |
| Turki 2013 (17) | rang 35-76 | NA | NA | NA | | | | N/A | NA | NA | NA | NA |
| Al-Badawi 2011 (18) | 56 (3.32) | 90 (100%) | NA | NA | | | | N/A | NA | NA | NA | Squamous cell carcinoma 78 (86.7%) Carcinoma in situ 5 (5.5%) Adenocarcinoma 7 (7.8%) |
| Alsbeih 2011 (19) | 46 (range 32-76)^#^ | NA | NA | NA | | | | N/A | NA | 7# | NA | AC: 18 (18%) SCC: 82 (82%) |
| Sait 2011 (20) | 48 (range 27–78) | NA | NA | NA | | | | N/A | NA | NA | NA | Squamous cell carcinoma 11/13 (84.61%) adenocarcinoma 2/13 (15.39%) |
| Al-Muammar 2007 (21) | NA | 75 (62.5%) | NA | 0 | 120 (100%) | 0 | 0 | N/A | 58 (48.3%) | NA | NA | NA |
| Gazzaz 2007 (22) | 41.97 (8.76) | NA | NA | NA | | | | N/A | NA | NA | NA | Abnormal cells with benign changes 0 (0%) ASC-US 4 (4%) AGC 2 (4%) Atypical endocervical cells with reactive changes 1 (1%) Atypical squamous cells 1 (1%) Koilocytotic changes as LSIL suggesting HPV infection 1 (1%) Cancer cells 0 (0%) |

*Data presented as mean (SD)

#Data presented as median (range)

**Table 3: Quality assessment of cross-sectional studies (n =11 studies)**

| Study ID | Selection Score | Justification | Comparability | Justification | Outcome | Justification | Overall (Based on total score) |
| --- | --- | --- | --- | --- | --- | --- | --- |
| Sait et al. 2024 (1) | **** | *The target population included women aged 30 to 65 who had been married for over 3 years, excluding those with prior cervical dysplasia, cervical cancer, or a history of hysterectomy. * * Participant recruitment for the large-scale HPV screening program involved various channels, including social media, primary health care clinics, collaborations with the Ministries of Health and Education, and female university employees. sample size wasn't caculated  * response rate was satisfactory | ** | * The study controls for the most important factor, HPV vaccination * The study controls for any additional factor. | * | * Factors associated with cumulative HPV positivity and clearance were analyzed using the independent t-test or the Mann-WhitneyU test for continuous variable, and chi-square test or Fisher’s exact test for categorical variables, as applicable. Univariate and multivariate logistic regression was used to analyze the independent factors influencing cumulative HPV positivity and clearance. The null hypothesis was rejected for a p-value of <0.05. | Low risk |
| Alshammari et al. 2022 (3) | **** | * 300 women transferred to a cytopathology laboratory at the maternity hospital in Al-Madinah, Saudi Arabia, between May 2020 and May 2021. The studied women were referred to do Pap smear because of gynecologic complaints. * response rate was satisfactory  ** Cytological samples were collected by scraping the transformation zone of the ectocervix, fixed in 95% ethyl alcohol for 15 minutes, then stained according to the Papanicolaou method (Pap. Method) as described elsewhere8 | ** | * The study controls for the most important factor, cytological change. * The study controls for any additional factor. | * | * The p-value < 0.05 is considered statistically significant contemplating 95% confidence interval. | Low risk |
| Ali 2019 (9) | **** | * female residents of KSA, Qatar, UAE, and Bahrain who attended gynecology outpatient clinics at health care facilities seeking care for a wide spectrum of gynecological complaints. * response rate was satisfactory  ** The cervical samples were collected by qualified gynecologists using the Cervex-Brush device (Rovers Medical Devices) according to the manufacturer’s instructions. | ** | * The study controls for the most important factor, cytological status. * The study controls for any additional factor. | ** | * Categorical variables were reported as the number of cases (percentage) and were compared using the Pearson chi-square test, whereas continuous variables were expressed as the mean ± SD and were compared using the independent Student t test or a 1-way analysis of variance, as appropriate. A 2-sided probability (P) value was used for all statistical analyses, and a P value <.05 was considered statistically significant. * The cytopathologists were unaware of the participants’ HR-HPV status, nor were the molecular biologists aware of the cervical cytology examination results. | Low risk |
| Mousa 2019 (10) | **** | *A convenience sample of self-collected vaginal swab samples were obtained from female participants attending the gynecological clinic in the period between October 2017 and April 2018 at a tertiary care center, Jeddah, Saudi Arabia. * response rate was satisfactory  ** All swabs were tested for HPV by polymerase chain reaction (PCR) and positive samples were then used to determine circulating genotypes by sequencing. | ** | * The study controls for the most important factor, age. * The study controls for any additional factor. | ** | * All participants answered a self-applied questionnaire after obtaining ethical approval from Unit of Biomedical Ethics Research committee at King Abdulaziz University Hospital in Jeddah. * Chi-square or Fisher exact tests were used for comparison of categoical data. A P<.05 values (two-sided test) was accepted as statistically significant. | Low risk |
| Alhamlan 2016 (12) | **** | * Women who attended the Primary Care Clinic (namely, Family Medicine) or the Obstetrics and Gynecology Clinic at King Faisal Specialist Hospital and Research Centre (KFSHRC) in Riyadh, Saudi Arabia, for routine cervical examinations from November 2013 to November2015were includedinthis2-year study. * response rate was satisfactory  ** Cervical specimens were collected using a cytobrush. Two cytobrushes were used to collect the specimens: the first was transferred into a vial containing liquid-based cytology transport medium | ** | * The study controls for the most important factor, age. * The study controls for any additional factor. | ** | * Questionnaires were completed by the enrolled participants and were collected by a clinical coordinator during the participants’ clinical visits. * All p-values reported were two-sided and were considered to be statistically significant at p < 0.05. | Low risk |
| Al-Ahdal 2014 (13) | **** | ** The gynecological examination of the subjects was performed according to the recommendations of the American Cancer Society. * response rate was satisfactory * The age of subjects ranged between 20 and 74 years (37 ± 9). | 0 | The study didn't control any factor | ** | * The Mantel-Haenszel (M-H) test of independence was used to assess the association between the categorical variables. M-H odds ratio estimates were produced with a 95% confidence interval (CI). Type I error rate was set at 5%. * demographic data sheets, from 519 normal women (Saudi citizens and non-Saudi legal residents) attending Obeid Specialized Hospital in Riyadh for routine checkups. | Moderate risk |
| AlObaid 2014 (14) | ***** | ** The study was conducted in accordance with the Declaration of Helsinki, good clinical practice guidelines, and local rules and regulations of the country. * Women aged ≥15 years undergoing routine gynecological examination and willing to provide a cervical sample were enrolled. * response rate was satisfactory  * To meet this objective, an estimated HPV prevalence ranging from 10 to 30% as previously reported. the required number of subjects ranged from 188 subjects for a 10% HPV prevalence to 450 subjects for a 30% prevalence, including an assumption of 10% of subjects non-evaluable. | ** | * The study controls for the most important factor, age. * The study controls for any additional factor. | ** | * An exploratory analysis was performed to assess the association between the HPV status and nationality (two sided Fisher’s exact test) and the adjusted odds ratio (adjusted for factors which are associated with the risk of HPV infection) was calculated using multivariate logistic regression model. * This multicenter, observational, cross-sectional, epidemiological study | Low risk |
| Bondagji 2013 (16) | **** | * all Saudi women attending gynecology clinics at King Abdulaziz University Hospital from March 2010 to January 2011 who fulfilled the inclusion criteria of being sexually active and of childbearing or postmenopausal age. ** The women then had the traditional Pap smear using the wet mount technique and the Hybrid Capture 2 (HC2) reagents and materials for HPV detection (Digene Corporation, USA). * response rate was satisfactory | 0 | The study didn't control any factor | * | * Data were collected and analyzed using SPSS statistical package version 16. | Moderate risk |
| Turki 2013 (17) | **** | * A woman was eligible for study subject if she was a gynecological outpatient with genital tract disease related symptom, was not presently pregnant; had not undergone a total uterus or cervix resection. ** DNA extraction was performed by using by Qiamp DNA mini kit (QIAGEN, Valencia, CA) with following manufacturer’s instructions. After extraction, all specimens were subjected to PCR amplification of the β-globin gene to serve as an internal control as described previously (Shadrina et al., 2007) and stored subsequently at –20o C until tested. * response rate was satisfactory | * | * The study controls for the most important factor, age. | 0 | No description of outcome assessment. | Moderate risk |
| Sait 2011 (20) | **** | ** After DNA extraction from the tissue, the HC2 an HPV DNA test, was performed according to the specifications of the manufacturer (Digene Corporation, Gaithersburg, MD), using the HPV DNA Test (Two-Probe Method). * response rate was satisfactory  *This pilot study was done on cervical biopsies performed on patients with cervical dysplasia and invasive disease, to test for HPV in the Saudi population. | * | * The study controls for the most important factor, cancer stage. | 0 | No description of outcome assessment. | Moderate risk |

**Table 2: Quality assessment of prospective and retrospective studies (n =11 studies)**

| **Study ID** | **Selection** | **Justification** | **Cofounder** | **Justification** | **Exposure** | **Justification** | **Overall (Based on total score)** |
| --- | --- | --- | --- | --- | --- | --- | --- |
| Faqih et al. 2023 (2) | ** | **This retrospective case-series observational study involved patients who visited King Fahad Medical City (KFMC) in Riyadh, Saudi Arabia, from January 2021 to December2022. All methods were performed in accordance with the Declaration of Helsinki and the relevant guidelines and regulations. | ** | * The study controls for the most important factor, cancer stage. * The study controls for any additional factor. | * | * Patients’ age, nationality, and Pap smear findings, including the pathological classification of cervical lesions detected during screening, along with the outcomes of HPV tests, were extracted from the clinical records. | Moderate risk |
| Alhamlan et al. 2021 (4) | ** | * The FFPE specimen blocks were processed using a standard method. * FFPE cervical specimens at various stages of cervical dysplasia and cancer archived from 2006 to 2016 were obtained from the Department of Pathology and Laboratory Medicine (CAP accredited) in King Faisal Specialist Hospital and Research Centre, the largest central and referral hospital in Saudi Arabia. | ** | * The study controls for the most important factor, histological classification. * The study controls for any additional factor. | * | * Demographic and clinical data were collected, including patient age, marital status, religion, nationality, and specimen pathologic results. | Moderate risk |
| Kussaibi et al. 2021 (5) | ** | * The results of 164 Saudi women coinvestigated for HR HPV along with Pap tests were retrieved from the archive. * all married women aged 21–65 years, every 3 years with or without HR HPV testing. Cervical Pap tests were collected at gynecology clinics in a commercially available liquid fixative vial. | * | * The study controls for the most important factor, histological classification. | * | * The results of 164 Saudi women coinvestigated for HR HPV along with Pap tests were retrieved from the archive. | Moderate risk |
| AlBabtain 2020 (6) | ** | * The record-based cross-sectional study was conducted at Department of Family Medicine and Polyclinics, King Faisal Specialist Hospital and Research Center (KFSH&RC), Riyadh, Saudi Arabia. * all the female patients from 21 to 65 years of age for pap smear results between January 2002 and January 2017. | 0 | The study didn't control any factor | * | * The record-based cross-sectional study was conducted at Department of Family Medicine and Polyclinics, King Faisal Specialist Hospital and Research Center (KFSH&RC), Riyadh, Saudi Arabia. | High risk |
| Alhamlan 2020 (7) | ** | * cervical specimens obtained from women attending outpatient clinics at the King Faisal Specialist Hospital and Research Centre (KFSHRC) in Riyadh, Saudi Arabia, were included in this study. * The inclusion criteria for participation in the study were women who were married, divorced, or widowed, and the exclusion criteria were women who were pregnant or virgin. | ** | * The study controls for the most important factor, histological classification. * The study controls for any additional factor. | * | * Demographic and clinical characteristics of the participants, including age, religion, marital status, and cervical cytology results were collected. | Moderate risk |
| Obeid 2020 (8) | ** | * The use of all specimens for the present study was previously approved by the Research Advisory Council (Ethics Committee) at King Faisal Specialist Hospital and Research Centre (KFSH & RC) (RAC 1005-033). * specimens were obtained, 618 from Pap testing using a PreservCyt brush (ThinPrep Pap Test Boxborough, MA, USA), and 316 specimens from archived biopsied, FFPE cervical specimens. | ** | * The study controls for the most important factor, histological classification. * The study controls for any additional factor. | * | * All data collected were stored and analyzed using SAS, version 9.4, and SPSS, version 24, software. | Moderate risk |
| Alsbeih 2017 (11) | ** | * The study used cervical tumor tissues obtained during routine diagnostic procedures of CxCa patients. The samples were processed anonymously. * CxCa patients treated between 1990 and 2012 in our tertiary hospital were reviewed, and 232 patients with histopathologically proven invasive tumors were included in the study. | ** | * The study controls for the most important factor, histological classification. * The study controls for any additional factor. | * | * CxCa patients treated between 1990 and 2012 in our tertiary hospital were reviewed, and 232 patients with histopathologically proven invasive tumors were included in the study. | Moderate risk |
| Al-Shabanah 2013 (15) | ** | * Genomic DNA was isolated from 100 FFPE ovarian carcinoma and their normal adjacent tissues. All samples were positive for β-globin gene amplification. * The patients’ mean age was 50 ± 11 years (range, 25–78 years). The patients’ age distribution was analyzed and the prevalence of HPV genotypes was detected among them. | 0 | The study didn't control any factor | 0 | No description of ascertainment of exposure. | High risk |
| Al-Badawi 2011 (18) | ** | * The archives of the Pathology Department at King Faisal Specialist Hospital and Research Center were searched for cases of cervical cancer and cervical carcinoma in situ. * All cases accessioned at the time of diagnosis between 1997 and 2007 and with available paraffin blocks. | * | * The study controls for the most important factor, age. | * | * The archives of the Pathology Department at King Faisal Specialist Hospital and Research Center were searched for cases of cervical cancer and cervical carcinoma in situ. | Moderate risk |
| Alsbeih 2011 (19) | ** | * One hundred paraffin-embedded cervical biopsies with histopathologically proven cervical cancer were collected from the pathology department.  * There were no restrictions on patients' age or histological type (squamous cell carcinoma, adenocarcinoma). | 0 | The study didn't control any factor | * | * The study was performed on archival materials of cervical cancer. | High risk |
| Al-Muammar 2007 (21) | ** | * during the routine gynocolgical examination of the primary care center, cervical scrapes were obtained from 120 women.  * all subjects were married | * | * The study controls for the most important factor, cytological change. | * | * patients were asked about their demographical data | Moderate risk |
| Gazzaz 2007 (22) | ** | * Digene’s HC2, HPV DNA test and PCR were used for HPV infection detection as a cancer cervical screening test in conjunction with the Pap test. * 100 sexually active women with an age range of 27-65 years visiting the Obstetric and Gynecology clinics at King Abdul-Aziz University Hospital (KAAUH), Jeddah, Saudi Arabia for different gynecological problems. | 0 | The study didn't control any factor | 0 | No description of ascertainment of exposure. | High risk |

**References**:

1. Sait KH, Anfinan NM, Sait HK, Basalamah HA. Human papillomavirus prevalence and dynamics: Insights from a 5-year population-based study in Jeddah, Kingdom of Saudi Arabia. *Saudi Med J* (2024) 45:252–260. doi: 10.15537/smj.2024.45.3.20230824

2. Faqih L, Alzamil L, Aldawood E, Alharbi S, Muzzaffar M, Moqnas A, Almajed H, Alghamdi A, Alotaibi M, Alhammadi S, et al. Prevalence of Human Papillomavirus Infection and Cervical Abnormalities among Women Attending a Tertiary Care Center in Saudi Arabia over 2 Years. *Trop Med Infect Dis* (2023) 8: doi: 10.3390/tropicalmed8120511

3. Alshammari FD, Alharbi SA, Humaida MI, Abdalhabib EK, Bealy MAB, Elkhalifa AEO, Agabeldour AA. Human Papillomavirus genotypes associated with cervical intraepithelial lesions among Saudi women. *Eur Rev Med Pharmacol Sci* (2022) 26:6367–6373. doi: 10.26355/eurrev_202209_29663

4. Alhamlan F, Obeid D, Khayat H, Asma T, Al-Badawi IA, Almutairi A, Almatrrouk S, Fageeh M, Bakhrbh M, Nassar M, et al. Prognostic impact of human papillomavirus infection on cervical dysplasia, cancer, and patient survival in Saudi Arabia: A 10-year retrospective analysis. *Ann Saudi Med* (2021) 41:350–360. doi: 10.5144/0256-4947.2021.350

5. Kussaibi H, Al Dossary R, Ahmed A, Muammar A, Aljohani R. Correlation of High-Risk HPV Genotypes with Pap Test Findings: A Retrospective Study in Eastern Province, Saudi Arabia. *Acta Cytol* (2021) 65:48–55. doi: 10.1159/000509669

6. AlBabtain FA, Hussain AN, Alsoghayer SA, Alwahbi OA, Almohaisen N, Alkhenizan AH. The yield of pap smears and its characteristics in a community based setting in Saudi Arabia. *Saudi Med J* (2020) 41:661–665. doi: 10.15537/SMJ.2020.6.25085

7. Alhamlan FS, Khayat HH, Obeid DA, Tulba AM, Baduwais TS, Alfageeh MB, Al-Ahdal MN. Clinical comparison of two human papillomavirus detection assays: GenoFlow and reverse line blot. *J Infect Dev Ctries* (2020) 14:97–103. doi: 10.3855/jidc.11769

8. Obeid DA, Almatrrouk SA, Khayat HH, Al-Muammer TA, Tulbah AM, Albadawi IA, Al-Ahdal MN, Alhamlan FS. Human papillomavirus type 16 and 18 viral loads as predictors associated with abnormal cervical cytology among women in Saudi Arabia. *Heliyon* (2020) 6:e03473. doi: 10.1016/j.heliyon.2020.e03473

9. Ali MAM, Bedair RN, Abd El Atti RM. Cervical high-risk human papillomavirus infection among women residing in the Gulf Cooperation Council countries: Prevalence, type-specific distribution, and correlation with cervical cytology. *Cancer Cytopathol* (2019) 127:567–577. doi: 10.1002/cncy.22165

10. Mousa M, Al-Amri SS, Degnah AA, Tolah AM, Abduljabbar HH, Oraif AM, Abduljabbar HS, Mirza AA, Azhar EI, Hashem AM. Prevalence of human papillomavirus in Jeddah, Saudi Arabia. *Ann Saudi Med* (2019) 39:403–409. doi: 10.5144/0256-4947.2019.403

11. Alsbeih GA, Al-Harbi NM, Bin Judia SS, Khoja HA, Shoukri MM, Tulbah AM. Reduced rate of human papillomavirus infection and genetic overtransmission of TP53 72C polymorphic variant lower cervical cancer incidence. *Cancer* (2017) 123:2459–2466. doi: 10.1002/cncr.30635

12. Alhamlan FS, Khayat HH, Ramisetty-Mikler S, Al-Muammar TA, Tulbah AM, Al-Badawi IA, Kurdi WI, Tulbah MI, Alkhenizan AA, Hussain AN, et al. Sociodemographic characteristics and sexual behavior as risk factors for human papillomavirus infection in Saudi Arabia. *Int J Infect Dis* (2016) 46:94–99. doi: 10.1016/j.ijid.2016.04.004

13. Al-Ahdal MN, Al-Arnous WK, Bohol MFF, Abuzaid SM, Shoukri MM, Elrady KS, Firdous N, Aliyan R, Taseer R, Al-Hazzani AA, et al. Human papillomaviruses in cervical specimens of women residing in Riyadh, Saudi Arabia: A hospital-based study. *J Infect Dev Ctries* (2014) 8:320–325. doi: 10.3855/jidc.4220

14. AlObaid A, Al-Badawi IA, Al-Kadri H, Gopala K, Kandeil W, Quint W, Al-Aker M, DeAntonio R. Human papillomavirus prevalence and type distribution among women attending routine gynecological examinations in Saudi Arabia. *BMC Infect Dis* (2014) 14:1–8. doi: 10.1186/s12879-014-0643-8

15. Al-Shabanah OA, Hafez MM, Hassan ZK, Sayed-Ahmed MM, Abozeed WN, Al-Rejaie SS, Alsheikh AA. Human papillomavirus genotyping and integration in ovarian cancer Saudi patients. *Virol J* (2013) 10:1–9. doi: 10.1186/1743-422X-10-343

16. Bondagji NS, Gazzaz FS, Sait K, Abdullah L. Prevalence of high-risk human papillomavirus infections in healthy Saudi women attending gynecologic clinics in the western region of Saudi Arabia. *Ann Saudi Med* (2013) 33:13–17. doi: 10.5144/0256-4947.2013.13

17. Turki R, Sait K, Anfinan N, Sohrab SS, Abuzenadah AM. Prevalence of human papillomavirus in women from Saudi Arabia. *Asian Pacific J Cancer Prev* (2013) 14:3177–3318. doi: 10.7314/APJCP.2013.14.5.3177

18. Al-Badawi IA, Al-Suwaine A, Al-Aker M, Asaad L, Alaidan A, Tulbah A, Bohol MF, Munkarah AR. Detection and genotyping of human papilloma virus in cervical cancer specimens from Saudi patients. *Int J Gynecol Cancer* (2011) 21:907–910. doi: 10.1097/IGC.0b013e318214219f

19. Alsbeih G, Ahmed R, Al-Harbi N, Venturina LA, Tulbah A, Balaraj K. Prevalence and genotypes’ distribution of human papillomavirus in invasive cervical cancer in Saudi Arabia. *Gynecol Oncol* (2011) 121:522–526. doi: 10.1016/j.ygyno.2011.01.033

20. Sait K, Gazzaz. Molecular tests to detect human papillomavirus infection in patients with cervical dysplasia and invasive cervical cancer in Saudi Arabia. *Pathol Lab Med Int* (2011)25. doi: 10.2147/plmi.s23059

21. Al-Muammar T, Al-Ahdal MN, Hassan A, Kessie G, Dela Cruz DM, Mohamed GE. Human papilloma virus-16/18 cervical infection among women attending a family medical clinic in Riyadh. *Ann Saudi Med* (2007) 27:1–5. doi: 10.5144/0256-4947.2007.1

22. Gazzaz FSB. Molecular testing of human papillomavirus in cervical specimens. *Saudi Med J* (2007) 28:1810–1818.
